# Interplay between epigenetics and metabolism in oncogenesis: mechanisms and therapeutic approaches

**DOI:** 10.1038/onc.2016.485

**Published:** 2017-01-16

**Authors:** C C Wong, Y Qian, J Yu

**Affiliations:** 1 Department of Medicine and Therapeutics, State Key Laboratory of Digestive Disease, Li Ka Shing Institute of Health Sciences, Shenzhen Research Institute, The Chinese University of Hong Kong, Hong Kong, China; 2 Department of Gastroenterology, Sir Run Run Shaw Hospital, School of Medicine, Zhejiang University, Hangzhou, China; 3 Institute of Gastroenterology, Zhejiang University, Hangzhou, China

## Abstract

Epigenetic and metabolic alterations in cancer cells are highly intertwined. Oncogene-driven metabolic rewiring modifies the epigenetic landscape via modulating the activities of DNA and histone modification enzymes at the metabolite level. Conversely, epigenetic mechanisms regulate the expression of metabolic genes, thereby altering the metabolome. Epigenetic-metabolomic interplay has a critical role in tumourigenesis by coordinately sustaining cell proliferation, metastasis and pluripotency. Understanding the link between epigenetics and metabolism could unravel novel molecular targets, whose intervention may lead to improvements in cancer treatment. In this review, we summarized the recent discoveries linking epigenetics and metabolism and their underlying roles in tumorigenesis; and highlighted the promising molecular targets, with an update on the development of small molecule or biologic inhibitors against these abnormalities in cancer.

## Introduction

It has been appreciated since the early days of cancer research that the metabolic profiles of tumor cells differ significantly from normal cells. Cancer cells have high metabolic demands and they utilize nutrients with an altered metabolic program to support their high proliferative rates and adapt to the hostile tumor microenvironment. Cancer cells could metabolize glucose via glycolysis to generate lactate, instead of oxidative phosphorylation (OXPHOS), even in the presence of normal oxygen levels.^1, 2, 3^ Although the process is less efficient compared with OXPHOS, glycolysis has a much higher turnover and provides intermediates for macromolecular biosynthesis and redox homeostasis. Apart from metabolizing glucose, cancer cells are addicted to glutamine. By means of a process known as glutaminolysis, cancer cells could divert a major fraction of glutamine to replenish the tricarboxylic acid (TCA) cycle.^4, 5, 6^ Hence, glutaminolysis supplies biosynthetic precursors for nucleotides, proteins and glutathione biosynthesis in tumorigenesis.^7, 8^

Oncogenic pathways have well-established roles in metabolic rewiring in human cancers. For instance, mutations in KRAS, PIK3CA, PTEN or AKT have been shown to hyperactivate mTOR-AKT pathway, which stimulates glycolysis via upregulation of glucose transporter 1 (GLUT1),^9, 10, 11^ and the phosphorylation of rate-limiting glycolytic enzymes, including hexokinases (HKs) and 6-phosphofructo-2-kinase/fructose-2,6-bisphosphatases (PFK2/FBPase2).^12, 13^ The oncogenic transcription factor MYC mediates the transcription of almost all the genes involved in glycolysis and glutaminolysis,^6, 14^ and it promotes shuttling of glycolytic intermediates to pentose phosphate pathway to generate large quantities of reduced nicotinamide adenine dinucleotide phosphate (NADPH) and promote macromolecule biosynthesis via the induction of pyruvate kinase isozymes M2 (PKM2).^15^ Numerous metabolic genes have also been identified as driver genes mutated in some cancers, such as isocitrate dehydrogenase 1 and 2 (IDH1/2) in gliomas^16^ and acute myeloid leukemia (AML),^17^ succinate dehydrogenase (SDH) in paragangliomas^18^ and fumarate hydratase (FH) in hereditary leiomyomatosis and renal cell cancer (HLRCC).^19^ Metabolic rewiring of cancer cells is considered as one of 10 hallmarks of cancer.^20^

Metabolic rewiring in cancer has profound effects on regulation of gene expression. Although metabolite profiles might have little impact on the genetic level, it appears that they have a fundamental role in epigenetic regulation of gene expression. Epigenetics refers to heritable changes in gene expression, which are not a consequence of alterations in the DNA sequence. Epigenetic regulation of gene expression can be highly plastic and responsive to various environmental clues.^21, 22, 23^ Epigenetics, which principally involved the chemical modification of DNA and histones, represents an innate mechanism that links nutritional status to gene expression. As such, metabolic rewiring could hijack the epigenome machinery in cancer cells to transmit a mitogenic gene expression profile.^24, 25, 26^ Reciprocally, epigenetic deregulation in cancer mediates, at least in part, to the altered expression of genes involved in cellular metabolism.

**Figure 1:**
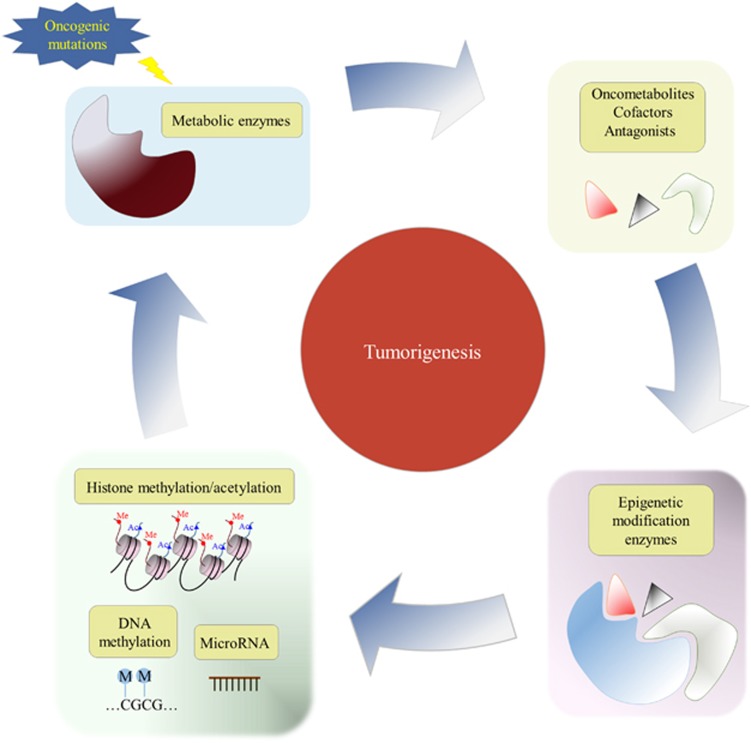
Crosstalks between epigenetics and metabolism in cancer development.

**Figure 2:**
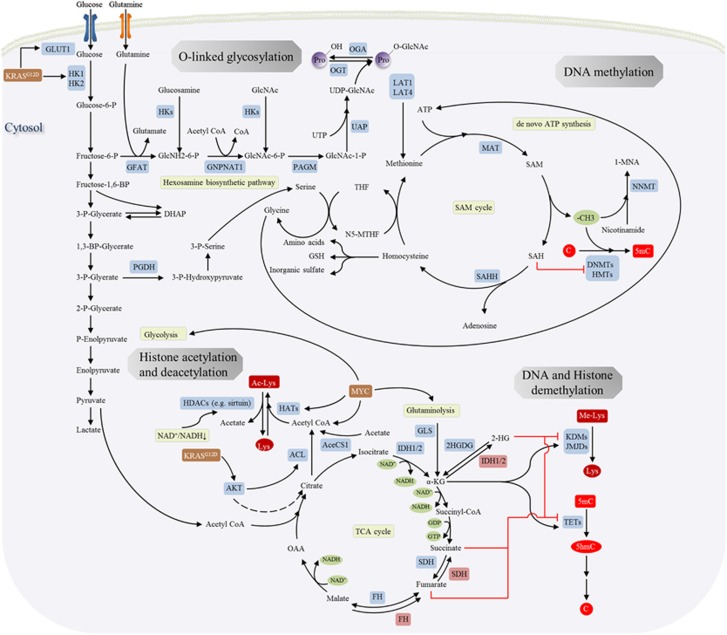
Effect of the tumor metabolome on the epigenetic processes such as histone acetylation, DNA methylation, DNA/histone demethylation, *N*-linked glycosylation in human cancers. An altered epigenetic regulation in turn contributes to deregulation of gene expression.

**Figure 3:**
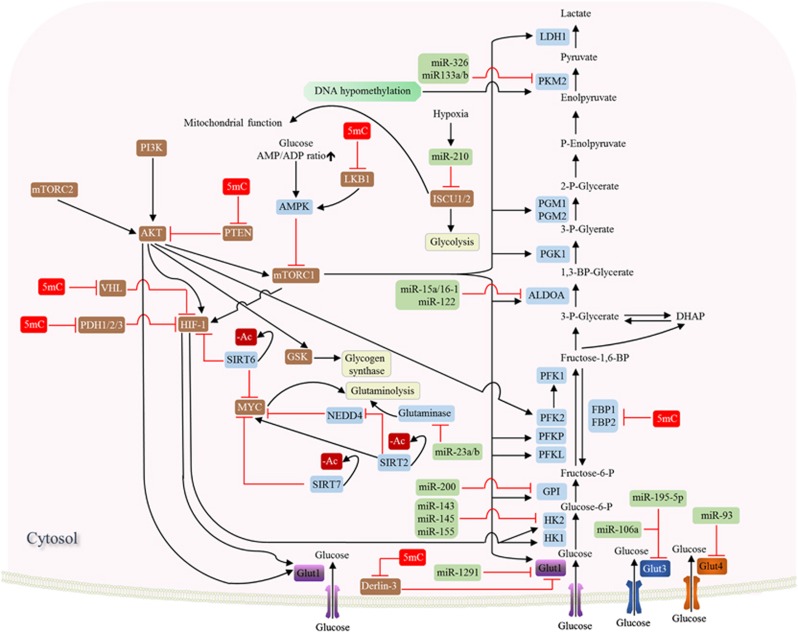
Effect of DNA hypermethylation (5mC), histone deacetylation (-Ac) and microRNA (miRs) on the expression of metabolic enzymes involved in glycolysis and glutaminolysis in cancer.

A four-way crosstalk exists between epigenetics and metabolism in cancer (Figure 1). Metabolic rewiring could affect the availability of cofactors required for epigenetic modification enzymes (1) and generate oncometabolites that act as agonists and/or antagonists for epigenetic modification enzymes (2), thus impacting the epigenetic landscape (Figure 2). On the other hand, epigenetic dysfunction modifies metabolism by directly affecting the expression of metabolic enzymes (3) and altering the signal transduction cascades involved in the control of cell metabolism (4) (Figure 3). In this review, we provide a summary of molecular mechanisms linking epigenetics and metabolism; and their underlying roles in tumorigenesis; highlight the potential molecular targets whose inhibition may abrogate these crosstalks and suppress tumorigenesis; and an outline of therapeutics against these potential drug targets.

## Effect of metabolic rewiring on epigenetic modification enzymes

### SAM/SAH ratio regulates DNA and histone methylation

DNA methylation is the most extensively studied epigenetic alteration in cancers. Promoter DNA methylation at CpG sites represses gene expression by impeding access to transcription factors and inhibition of RNA polymerase II.^27, 28, 29^ In cancer, aberrant DNA methylation is typically observed in the promoter regions of various tumor suppressor genes and microRNAs,^30, 31, 32, 33, 34, 35, 36, 37, 38^ leading to their transcriptional silence. DNA methylation is mediated by DNA methyltransferases, which catalyze the covalent addition of a methyl group to cytosine to form 5-methylcytosine (5 mC). All DNMT isoforms, DNMT1, DNMT3A and DNMT3B, are overexpressed in cancers.^39^ Methylation markers on lysine residues in histone proteins also have a key role in regulating chromatin structure and gene transcription. Multiple lysine residues (H3K4, H3K9, H3K27, H3K36, H3K79 and so on) may be mono-, di- or tri-methylated, giving rise to a very complex histone methylation code.^40^ Histone methyltransferases (HMTs) that mediate histone-lysine methylation consist of two enzyme families, SET-domain (SETD) containing and Dot1-like (DOT1L) proteins.^41^

DNMTs and HMTs utilize a common activated methyl donor for methyltransferase activity: *S*-adenosylmethionine (SAM). SAM is a product of one-carbon metabolism cycle and is synthesized by methionine adenosyltransferase (MAT) using methionine and ATP as substrates. Donation of methyl group from SAM invariably releases *S-*adenosyl-homocysteine (SAH) as the product and the latter is a potent inhibitor of methyltransferase such as DNMTs and HMTs. Hence, the SAM/SAH ratio dictates methyltransferase activity *in vivo*. SAH is physiologically maintained at low levels via hydrolysis to homocysteine, which can be recycled to methionine via the transfer of a methyl group from 5-methyl-tetrahydrofolate. Alternatively, homocysteine can be catabolized to give amino acids, glutathione and inorganic sulfate. Changes in SAM/SAH ratio and one-carbon cycle will thus modulate the activity of DNMTs and HMTs.^42^

An excess supply of SAM might contribute to DNA hypermethylation at CpG sites and inappropriate gene silencing. Glycine *N*-methyltransferase (GNMT) deficiency is a rare genetic condition leading to SAM over-production.^43^ The genetic knockout of Gnmt in mice increased hepatic SAM by over 40-fold.^43^ Moreover, *Gnmt* knockout mice demonstrated promoter methylation of tumor suppressor genes such as RASSF1 and SOCS2, which led to their transcriptional silencing.^44^ As a consequence, *Gnmt* knockout was associated with activation of oncogenic pathways and an increased incidence of hepatocellular carcinoma.^44^ Cancer cells have also been shown to boost SAM availability via promoting one-carbon metabolism. Cancer cells could directly increase the uptake of methionine through the overexpression of amino-acid transporters LAT1 and LAT4 (SLC7A5/SLC43A2).^45, 46^ Alternatively, overexpression of 3-phosphoglycerate dehydrogenase (PGDH) diverts glycolysis intermediates to the serine-glycine biosynthesis pathway.^47, 48^ Serine participates in one-carbon metabolism through donation of its side chain to tetrahydrofolate to drive the folate cycle, which in turn recycles methionine from homocysteine. Serine also supports SAM synthesis from methionine through *de novo* ATP synthesis, a major contributor to the functional ATP pool in cancer cells.^49^

Alterations in SAM/SAH ratio also profoundly affect aberrant histone methylation in cancers. Nicotinamide *N*-methyl-transferase (NNMT) catalyzes the conversion of nicotinamide to 1-methylnicotinamide (1-MNA) using SAM as methyl donor. NNMT is overexpressed in a variety of cancers, including lung, liver, kidney bladder and colon cancers and exerts an oncogenic effect.^50, 51, 52, 53^ Recently, NNMT expression was found to be upregulated in human embryonic stem cells, and NNMT is indispensable for the maintenance for pluripotency. NNMT serves as a sink for SAM, severely depleting cellular SAM pool, resulting in >50% reduction in the SAM/SAH ratio and making SAM unavailable for HMTs.^54^ As a consequence, cell lines overexpressing NNMT showed a substantial decrease in histone methylation marks at H3K4, H3K9, H3K27 and H4K20. Altered histone methylation further regulated key signaling pathways associated with acquisition of a more aggressive/pluripotent phenotype. Conversely, knockdown of NNMT increased histone methylation. However, DNA methylation was not affected by NNMT overexpression or knockdown. The apparent discrepancy between DNA and histone methylation may arise from varying *K*_m_ values of DNMTs and HMTs for SAM. HMTs possess high *K*_m_ values for SAM, thereby conferring a higher sensitivity to changes in SAM levels.

SAM, as an activated methyl donor for DNMTs and HMTs, has a major impact on the epigenomic landscape. Given the myriad of processes that are affected by histone and DNA methylation, and the diversity of the downstream signaling pathways involved, deregulation of SAM levels in cancers likely has a context-dependent effect, and much remains to be explored.

### TCA cycle metabolites modulate DNA and histone demethylation

The dynamics of DNA and histone methylation is additionally regulated by the activity of DNA and histone demethylases, respectively. Methylated cytosine residues are demethylated in two sequential steps, involving oxidation of 5-methyl-cytosine (5-mC) to 5-hydroxymethyl-cytosine (5-hmC), catalyzed by the 10–11 translocation (TETs) family of proteins,^55, 56, 57^ followed by reversion to cytosine through oxidation and base excision repair by thymine DNA glycosylase (TDG).^58^ TETs are putative tumor suppressors. Frequent inactivating TET2 mutations have been detected in myeloid lineage malignancies^59^ and downregulation of TETs have been observed in several human cancers.^60, 61, 62^ Therefore, hyperactive methylation and deactivated demethylation machinery work in conjunction to induce promoter DNA hypermethylation in cancers. Demethylation of histone lysine marks is mediated by flavin-dependent Histone Lysine Demethylases that consist of lysine-specific protein demethylases (KDM1) family and jumonji C-domain-containing (JMJD) enzymes. The role of histone demethylases in cancer is less clear-cut.^63^ In some cases, histone demethylases are downregulated by gene mutations or deletions in cancers, but in others they can be amplified, such as JMJD2C^64^ and lysine-specific protein demethylases LSD1.^65^ TETs and JMJDs both belong to α-ketoglutarate (α-KG)-dependent dioxygenases that requires α-KG as a cofactor and is competitively inhibited TCA cycle intermediates such as succinate and fumarate.^66^ Cancer cells with mutations in metabolic genes may gain the ability to accumulate or synthesize metabolites, such as 2-hydroxylglutarate (2-HG), succinate and fumarate.

#### 2-hydroxyglutarate

Mutations in the metabolic enzymes isocitrate dehydrogenase (IDH) isoforms IDH1 and IDH2 are common in gliomas,^16, 67^ AML^17, 68, 69^ and angioimmunoblastic T-cell lymphoma.^70^ IDH1/2 are Nicotinamide Adenine Dinucleotide Phosphate (NADP)^+^-dependent metabolic enzymes that participate in the TCA cycle, catalyzing a two-step reaction for oxidative decarboxylation of isocitrate to α-KG.^71^ Mutations in IDH1/2 occur at substrate binding sites (IDH1: R132; IDH2: R140/172). Mutant IDH1/2 possess oncogenic properties, and their ectopic expression enhanced cancer cell proliferation, colony formation and inhibits cellular differentiation *in vitro*.^69, 72, 73^ These mutations abrogate the ability of IDH1/2 to synthesize isocitrate from α-KG but are accompanied by the gain-of-function conversion of α-KG to 2-HG.^72^ 2-HG is pivotal to the functional effect of mutant IDH1/2. This oncometabolite accumulates to very high levels (5 to 35 mM) in mutant IDH1/2 tumors. 2-HG is structurally similar to α-KG and it acts as a competitive antagonist. Thus, 2-HG inhibits activity of α-KG dependent dioxygenases, such as TETs and JMJDs, which have broad implications for the regulation of epigenome.^74^ Apart from tumors with mutant IDH1/2, increased 2-HG have also been reported in breast cancer^75^ and renal cancer (L-enantiomer),^76^ which is associated with activation of MYC and L-2-hydroxyglutarate dehydrogenase (L2HGDG) deficiency, respectively. The former promotes glutaminolysis and 2-HG production via wild type IDH2; while inactivation of L2HGDG prevents conversion of 2-HG back to α-KG.^77^

TET1/2-mediated conversion of 5mC to 5hmC is a relevant target of 2-HG.^17, 74^ *In vitro* enzymatic assays with TET1/2 revealed that 2-HG behaves as a competitive inhibitor.^78, 79^ Its inhibitory effect was especially pronounced for TET2, with 33% and 83% at 10 and 50 mM, respectively. Either introduction of mutant IDH1/2 or 2-HG abrogated TET1/2-mediated formation of 5-hmC in human cell lines. Moreover, ectopic expression of mutant IDH1^R132H^ into primary human astrocytes is sufficient to produce a CpG island methylator phenotype (CIMP) by inducing hypermethylation in a large number of genes. In human patients, IDH1/2 mutations in glioma or AML define distinct patient subgroups associated with CIMP.^80, 81^ DNA hypermethylation induced by 2-HG is reversible, and therefore represents a viable therapeutic target in IDH1/2-mutant cancers.^17^

2-HG levels in IDH1/2 mutant tumors also have implications for histone demethylase activity.^78^ 2-HG strongly inhibited several histone demethylases (JMJD2A/KDM4A, JMJD2C/KDM4C and JHDM1A/KDM2A) as compared to other dioxygenases. Other studies additionally identified 2-HG as a histone demethylases KDM7A inhibitor^73, 79^ by binding to catalytic core and competing with α-KG. In U-87MG (human glioma cells), 2-HG or overexpression of mutant IDH1 increased H3K9, H3K27 and H3K79 dimethylation and H3K4 trimethylation.^79^ Knockin of IDH1^R132H^ in haematopoietic cells was associated with increased dimethylation of H3K79 and trimethylation of H3K4, H3K9, H3K27 and H3K36.^74^ Alterations in these methylated histone marks, in particular H3K9 trimethylation, were found to promote pluripotency and inhibit differentiation.^73^ Human primary glioma with mutant IDH1 had elevated H3K79 dimethylation levels compared with those with wild type;^73^ oligodendroglioma patients with mutations in IDH1 also had higher H3K9me3 compared with those with wild-type IDH1/2.^82^ Given a large number of JMJDs enzymes and their diverse substrate specificities, more studies are required to unravel the full spectrum of histone methylation induced by 2-HG and its biological significance.

#### Succinate and fumarate

Inactivating mutations in TCA cycle enzymes fumarate hydratase (FH) and succinate dehydrogenase (SDH) are driver mutations in a subset of human cancers and they mediate epigenetic reprogramming.^83^ SDH mutations are present in gastrointestinal stromal tumors (GISTs), renal cell carcinoma, paraganglioma and pheochromocytoma. ^84, 85, 86, 87, 88^ SDH consists four subunits (SDHA, SDHB, SDHC and SDHD) and it catalyzes oxidation of succinate to fumarate. Mutations in any of the four subunits can inactivate the SDH complex, leading marked accumulation of succinate. Mutations in FH have been detected in HLRCC.^19^ Mitochondrial FH mediates the reversible conversion between fumarate and malate, and the loss-of-function mutation of FH resulted in high levels of fumarate.

Recent data have shed new light on the mechanisms of the tumor suppressor effect of FH and SDH. Both succinate and fumarate behave as α-KG competitive antagonists for inhibiting TETs and JMJDs. Both of these metabolites inhibited TETs-catalyzed hydroxylation of 5mC and the activity of histone demethylases KDM2A and KDM4A.^66^ Ectopic expression of FH and SDH mutants recapitulated the effect of fumarate and succinate. Furthermore, mouse chromaffin cells with genetic knockout of Sdhb exhibited a methylator phenotype, with an increased 5-mC/5-hmC ratio and enhanced histone methylation at H3K9, H3K27, and H3K27.^89^ Epigenetic dysregulation in Sdhb knockout cells triggered a transcriptional program that downregulated genes associated with the suppression of metastasis, leading to increased cell invasiveness. Consistent with *in vitro* data, a deregulated epigenomic landscape is frequently observed in FH or SDH mutant tumors. Gastrointestinal stoma tumors (GISTs) harboring mutant SDH have genomic DNA methylation an order of a magnitude greater than c-Kit-mutated GISTs.^90^ Genomic hypermethylation was also observed in patients with SDH-mutant hereditary paraganglioma and pheochromocytoma. Moreover, paraganglioma patients with SDH or FH-deficiency associated DNA CIMP had a much worse prognosis compared with other molecular subtypes, indicating that epigenetic dysregulation in SDH or FH-mutant patients contributes to tumor development and progression.^89^ Thus, genetic mutations in FH and SDH can lead to accumulation of fumarate and succinate, respectively, which drives tumorigenesis via epigenetic deregulation.

### Acetyl-CoA and NAD^+^ influence histone acetylation

Histone acetylation involves the addition of an acetyl group to lysine residues. Histone acetylation is dynamically regulated by opposing actions of histone acetyltransferases (HATs) and histone deacetylases (HDACs) that catalyze the addition and removal of the acetyl group, respectively. HATs are divided into GCN5/PCAF, p300/CBP and MYST (MOZ, Ybf2/Sas3, Sas2, Tip60) families, whereas HDACs are classified into four groups: the zinc-dependent class I, II and IV and NAD^+^-dependent class III HDACs (also known as sirtuins). Histone acetylation decreases the electronic interaction between histones and negatively charged DNA, which is associated with a more open chromatin structure and active gene transcription ^91^. HDAC-mediated histone deactylation has well-recognized roles in cancers via transcriptional repression of tumor suppressor genes.^92, 93^ While some HATs are also putative tumor suppressors and inactivating mutations in p300/CBP have been identified in breast, colorectal and gastric cancers,^94, 95^ several fusion genes that involve HATs, such as MLL-CBP^96^ and MOZ-TIF2^97^ behaves as oncogenic factors in hematological malignancies.

#### Acetyl-CoA

Acetyl-CoA is an important molecule in intermediary metabolism. It fuels the TCA cycle and it is at the crossroads of glycolysis, glutaminolysis and β-oxidation of fatty acids in mitochondria. Cytosolic and nuclear acetyl-CoA levels are maintained by two metabolic pathways, its direct synthesis from acetate and CoA by acetyl-CoA synthetase short-chain family 1 (AceCS1); and the conversion from citrate to acetyl-CoA by ATP citrate lyase (ACL).^98^ Acetyl-CoA is utilized extensively as a cofactor for enzymes that catalyze the transfer of an acetyl group, including HATs that utilize the acetyl group of acetyl-CoA to form ε-*N*-acetyl-lysine. Intracellular concentrations of acetyl-CoA can vary roughly ~10-fold under normal physiological conditions and it falls within the *K*_m_ range of HATs. Histone acetylation activity is thus dynamically regulated by availability of acetyl CoA.

Both absolute acetyl-CoA and the ratio of acetyl-CoA to coenzyme A have been shown to regulate histone acetylation in cancer.^99, 100^ Availability of acetyl-CoA for HATs is primarily modulated by 1) ACL expression; and 2) the availability of citrate as a substrate for ACL. ACL protein expression is localized to the nucleus and its activity contributes to nuclear specific acetyl-CoA pool.^100^ ACL silencing in HCT116 cells suppressed histone acetylation for all core histones, whereas the knockdown of AceCS1 had no effect. Non-histone protein acetylation was unaltered, suggesting that ACL-derived acetyl-CoA has a specific role in regulating histone acetylation. Cancer cells often overexpress ACL^101^ and this probably contributes to nuclear acetyl-CoA pool that is necessary for histone acetylation and expression of glycolytic enzymes. Oncogene-driven metabolic reprogramming in cancers also promotes a high level of glycolytic flux and mitochondrial production of citrate, which translocates to the cytosol and nucleus. In mouse pancreas, expression of constitutive activated KRAS^G12D^ allele resulted in high histone H3 and H4 acetylation in pancreatic adenocarcinoma.^99^ KRAS^G12D^ dependent activation of AKT promoted nuclear acetyl-CoA accumulation via (1) induction of glycolysis, leading to overproduction of citrate; and (2) ACL phosphorylation and activation. Indeed, constitutively active AKT induced a rapid and pronounced rise histone acetylation in cancer cells,^99^ further confirming its role in mediating histone acetylation.

Another player involved in histone acetylation is MYC. MYC has been shown to upregulate the expression of HAT-GCN5, which induced mono-, di-, tri-, and tetra-acetylation of histone H4 N-terminal.^102^ Besides, MYC mediates gene expression of metabolic enzymes linked to acetyl-CoA synthesis, including glycolysis and glutaminolysis. In isogenic rat fibroblasts with Myc^−/−^ or Myc^+/+^, it was demonstrated that Myc increased the mitochondrial export of acetyl-groups and a majority of these acetyl equivalents ended up in histone H4-K16.^103^ These data emphasizes the role of Myc in modulating the gene expression of HATs and the availability of acetyl-CoA to support histone acetylation in response to proliferative signals. Hence, multiple oncogenic signals contribute to increased histone acetylation to regulate gene expression.

#### NAD^+^

NAD^+^ is essential for the deacetylation activity of sirtuins, a subgroup of HDAC, and changes in NAD^+^/NADH ratio is thought to positively regulate activity of sirtuins. NAD^+^/NADH ratio is closely associated with energy status in cells. When energy is plentiful, NAD^+^/NADH ratio drops; while NAD^+^ level can be induced in nutrient deprived conditions. Hence, through sensing of NAD^+^/NADH levels, sirtuins serves as a link between energy status and regulation of gene expression.^104^ High glycolytic activity in cancers often generates a low NAD^+^/NADH ratio that is inhibitory for sirtuins. Repressed sirtuins, together with increased HATs activity induced by acetyl-CoA, may contribute to histone hyperacetylation and aberrant gene transcription. More studies are needed to understand the role of metabolic status and sirtuin activation in cancer.

### Hexosamine biosynthetic pathway promotes protein glycosylation

*O*-GlcNAcylation is one of the most common post-translational modifications in eukaryotic cells, via attachment of *O*-linked β-D-*N*-acetylglucosamine (*O*-GlcNAc) to Ser/Thr residue. *O*-GlcNAcylation is regulated by competing actions of *O*-GlcNAc transferase (OGT) and *O*-GlcNAcase (OGA), which, respectively, catalyzes addition and removal of *O*-GlcNAc from proteins. Recent evidence indicate that modifications of all core histone proteins by *O*-GlcNAc constitute part of the histone code.^105, 106, 107, 108^ OGT has been shown to coordinate with TETs to modulate *O*-GlcNAcylation of histone H2B to activate gene transcription;^109, 110^ whereas its association with EZH2 in polycomb repressive complex 2 regulates H3K27 me3 to silence tumor suppressor genes.^111^ Alternatively, OGT activates gene transcription via *O*-GlcNAcylation of C-terminal domain of RNA polymerase II.^112^ Elevated OGT expression and protein hyper-*O*-GlcNAcylation is a common feature in human cancers.^113^ Cell metabolism has a crucial role in *O*-GlcNAcylation via modulating the biosynthesis of uridine diphosphate β-D-*N*-acetylglucosamine (UDP-GlcNAc), the activated substrate for *O*-GlcNAcylation.

#### *O*-GlcNAc

*O*-GlcNAc is synthesized via the hexosamine biosynthetic pathway (HBP). In this pathway, glucose entering glycolysis is first metabolized to glucose-6-phosphate (glucose-6-P) and then fructose-6-phosphate (fructose-6-P). About 2–5% of the fructose-6-P will be diverted to fructose-6-P amidotransferase (GFAT), the first and rate limiting enzyme of HBP. GFAT converts fructose-6-P to glucosamine-6-phosphate (GlcNH_2_-6-P) utilizing an amine group from glutamine. A sequence of reactions then adds an acetyl group from acetyl-CoA and UDP from UTP results in production of UDP-GlcNAc. Hence, HBP integrates substrates from carbohydrate, amino acid, fat and nucleotide metabolism. Glycolysis and supply of glutamine have a pivotal role in UDP-GlcNAc synthesis, as they are obligatory for the rate limiting reaction catalyzed by GFAT. Cancer cells frequently demonstrate the upregulation of HBP, which is in turn associated with aberrant *O*-GlcNAcylation and increased malignant behavior.^114, 115, 116, 117, 118^ The upregulation of HBP in cancer cells is primarily driven by an increased glucose uptake and metabolism. In KRAS^G12D^-driven pancreatic cancer, KRAS^G12D^ promotes glucose utilization via increased expression of glucose transporter 1 (*Glut1)*, hexokinase 1 (*Hk1)* and hexokinase 1 (*Hk2*), which induce metabolic flux through GFAT and protein *O*-GlcNAcylation.^30^ KRAS^G12D^ inactivation, in contrast, downregulated global protein glycosylation, suggesting that KRAS^G12D^-mediated glucose utilization via HBP is essential for *O*-GlcNAcylation. In another study, tumor hypoxia was found to co-ordinately upregulation of glucose and glutamine utilization via HBP, which increased protein *O*-GlcNAcylation required for tumor survival.^119^ Conversely, glucose deprivation suppressed *O*-GlcNAcylation and cell growth, an effect reversed by addition of *N*-acetylglucosamine, a HBP substrate. These studies confirm the role of accelerated glucose metabolic in protein *O*-GlcNAcylation in cancer cells.

## Epigenetic regulation of metabolic genes expression in cancers

### DNA methylation

DNA methylation has been shown to modulate the expression of metabolic genes directly by regulating their transcription or indirectly via dysregulation of oncogenic cascades (for example, AKT, AMPK and HIF). DNA methylation mediates silence of fructose-1,6-bisphosphatase 1 (FBP1) and fructose-1,6-bisphosphatase 2 (FBP2) via promoter methylation in breast, gastric, liver and colorectal cancers.^120, 121, 122, 123^ FBP1 and FBP2 are rate limiting enzymes for gluconeogenesis that antagonize glycolysis, and their decreased expression promotes glycolytic flux for driving macromolecules biosynthesis and ATP production. DNA methylation also mediates overexpression of glucose transporter 1 (GLUT1) by epigenetic loss of Derlin-3, a key gene involved in the proteasomal degradation of GLUT1.^124^ Conversely, promoter hypomethylation contributes to upregulation of pyruvate kinase ioszyme 2 (PKM2) in multiple cancer types.^125^ PKM2 is a less active isomer that drives glucose flux towards macromolecules biosynthesis and is the predominant isoform in actively proliferating cells. DNA methylation also drives transcriptional silencing of tumor suppressor genes involved in signaling cascades linked to tumor metabolism. PI3K/AKT/mTOR and HIF-1 signaling are central activators of glycolysis and cancer-related metabolism. Multiple tumor suppressors that repress PI3K/AKT/mTOR and HIF-1 signaling are epigenetically silenced by promoter hypermethylation, including PTEN^126, 127, 128, 129, 130^, LKB1,^131, 132^ VHL^133, 134, 135, 136^ and prolyl hydroxylases (PHD1/2/3).^137, 138^ Hence, differential DNA methylation significantly contributes to glycolytic phenotype in human cancers.

### Histone modifications

Among the histone modification enzymes, the role of sirtuins (SIRTs) in regulating cell metabolism has been most extensively investigated. SIRT6 regulates glucose homeostasis by modulating histone acetylation.^139^ SIRT6 interacts directly with HIF1 and MYC, and it functions as a co-repressor through histone deacetylation, thereby inhibiting transcription.^139, 140, 141, 142^ SIRT6 hence acts as a tumor suppressor by repressing HIF-dependent glycolytic switch and MYC-dependent ribosome biogenesis and glutaminolysis. SIRT6 knockout induced a shift towards a ‘glycolytic phenotype’ and promoted cancer formation and aggressiveness. Consistent with its tumor suppressive role, frequent deletions in SIRT6 have been detected in cancer cell lines and colon, pancreatic and hepatocellular cancers.^143^ SIRT7 is another sirtuin that directly interacts with MYC.^144, 145^ SIRT7 possesses selective catalytic activity towards H3K18Ac. As H3K18 deacetylation is a repressive mark, it is not surprising that SIRT7 opposes MYC- dependent gene regulation and thus suppresses MYC-mediated metabolic alternations. In contrast to SIRT6/7, SIRT2 promotes deregulated metabolism through indirectly stabilizing MYC.^146^ SIRT2 deacetylases histone at H4K16, leading to suppressed expression of ubiquitin-protein ligase NEDD4. NEDD4 is a negative regulator of MYC by targeting it for ubiquitination and degradation. Critically, SIRT2 is itself upregulated by MYC in cancer cell lines; it constitutes a positive-feedback loop that promotes MYC-dependent transcription and oncogenesis. Given the myriad of histone modifications that contributes to gene regulation, much remains to be understood with regards to the role of histone code on metabolic reprogramming in cancer.

### miRNA

MicroRNAs (miRNAs) regulate expression of genes involved in diverse cellular functions. Several aspects of cell metabolism are regulated by miRNAs, including glycolysis and mitochondrial TCA cycle, thereby contributing to the Warburg’s effect. miRNAs also have a major impact on the signal transduction via PI3K/AKT, HIF1 and Myc that contribute to the metabolic phenotype in human cancers. miRNAs regulates the expression of numerous genes taking part in glucose uptake, including miR-1291 for GLUT1,^147^ miR-195-5p and miR-106a for GLUT3,^148, 149^ and miR-93 for GLUT4.^150^ as well as glycolysis, such as miR-143, miR-145 and miR-155 for hexokinase 2 (HK2),^151, 152, 153, 154, 155, 156^ miR-200 for glucose-6-P isomerase (GPI),^157^ miR-15a/16-1 and miR-122 for aldolase A (ALDOA),^158^ miR-326 and miR133a/b for PKM2.^159, 160^ Glutaminolysis is also targeted by miRNAs (miR-23a/b) via glutaminase.^161^ Deregulation of aforementioned miRNAs has been reported in cancers and they contribute to increased glycolysis and glutaminolysis in cancers. Moreover, miR-210, by repressing iron–sulfur cluster assembly proteins (ISCU1/2), inhibits mitochondrial function. miR-210 therefore favors a shift towards a glycolytic phenotype and lactate production, which is critical for adaptation to hypoxic tumor microenvironment.^162^ miRNAs also have a profound effect on signal transduction. PI3K/AKT/mTOR, LKB1/ AMPK, MYC, and HIF1 signaling cascades have all been shown to be regulated by miRNAs. Hence, the dysregulation of metabolic signaling pathways by miRNAs additionally contributes to altered metabolism in cancers.

## Therapeutic opportunities targeting epigenetic-metabolism crosstalks in cancer

### Reversal of epigenetic dysregulation by targeting cancer metabolism

#### Glycolysis inhibitors

Accelerated glycolysis in cancer contributes to histone acetylation via citrate and acetyl CoA. Histone acetylation in cells is regulated by glucose flux in a dose-dependent manner^163^ and elevated glycolysis in cancer is associated with global histone hyperacetylation.^164^ Inhibition of glycolysis holds promise for modulating histone acetylation. 2-Deoxyglucose (2-DG) is a glucose analog that is transported to cells and metabolized by hexokinase (HK) to form 2-DG-P. 2-DG-P cannot be further metabolized by phosphohexose isomerase,^165^ leading to the feedback inhibition of hexokinase, a rate-limiting enzyme for glycolysis. Treatment with 2-DG significantly suppresses acetyl-CoA levels, and the acetylation of histone H3, H4, H2A and H2B in multiple cancer cell lines.^164^ The reductions in global histone acetylation by 2-DG compromise DNA repair and sensitize cancer cells to DNA-damaging therapeutics. Acetyl-CoA is also required for maintenance of pluripotency through histone acetylation and glycolysis inhibition by 2-DG or 3-bromopyruvate (BrPA, a GAPDH inhibitor), which was found to induce differentiation in embryonic stem cells.^166^ Hence, glycolysis represents a viable target for modulating histone acetylation.

#### Glutaminolysis inhibitors

Glutaminolysis is frequently elevated in cancer, and accumulating evidence indicates that its inhibition is effective for targeting glutamine-addicted cancers.^5^ Glutaminase (GLS), which catalyzes the deamination of glutamine to glutamate, is the most extensively studied drug target in this pathway. Several inhibitors, such as bis-2-(5-phenylacetamido-1,2,4-thiadiazol-2-yl) ethyl sulfide (BPTES),^167^ compound 968^168^ and CB-839^167^ have been characterized, and CB-839 is currently undergoing Phase I dose escalation trials in solid and hematological malignancies (Table 1). Glutaminolysis generates α-KG, TCA cycle intermediates and acetyl-CoA, which in turn influence epigenetic status. Indeed, treatment of breast cancer cells with compound 968 significantly altered histone H4K16 acetylation and histone H3K4 methylation, leading to downregulation of numerous cancer-related genes.^169, 170^ Using an unbiased small molecule screen, Elhammali *et al.*^171^ unravelled Zaprinast, a phosphodiesterase 5 inhibitor, as a potent inhibitor of mutant IDH1^R132C^-mediated 2-HG biosynthesis in HT1080 cells. Surprisingly, Zaprinast did not target mutant IDH1, but instead it suppressed GLS. GLS-mediated glutaminolysis is essential for maintaining supply of α-KG, upstream of IDH1/2 metabolism of α-KG to 2-HG. As a consequence, Zaprinast treatment resulted in a marked reduction in histone H3K9me2/3 methylation. These studies highlight potential utility of GLS inhibitors in the reversal of epigenetic dysregulation in cancer, especially in the context of IDH1/2 mutations.

#### IDH1/2 inhibitors

Inhibition of IDH1/2 has been pursued as a strategy to suppress the production of oncometabolite 2-HG. Rohle *et al.*^172^ described the first selective inhibitor (AGI-5198) of mutant IDH1,^R132H^ which selectively inhibited mutant IDH1 (IC_50_=70 nM), but not wild-type IDH1 or any of the IDH2 isoforms (IC_50_>100 μM). In IDH1-mutant glioma cells, AGI-5198 inhibited 2-HG production and cell growth *in vitro* and *in vivo*. AGI-5198 induced demethylation of H3K9me3 and H3K27me3, whilst it had no effect on DNA methylation. AGI-5198 also demonstrated anticancer activity in human chondrosarcoma cells harboring IDH1 mutations.^173^ Subsequently, novel mutant IDH1^R132H^ inhibitors has been reported, such as AG-120, AG-881, ML309,^174^ 2-(3-trifluoromethylphenyl)isothioazol-3(2H)-one,^175^ bis imidazole phenol (cpd 1),^176^ 1-hydroxypyridin-2-one compounds^177^ and GSK321 and GSK864^178^ (Table 1). These drugs inhibited mutant IDH1^R132H^ in nanomolar range and exhibited promising selectivity over wild type IDH1.

AG-221 is a first-in-class inhibitor of mutant IDH2^179^ (Table 1). AG-221 was found to suppress 2-hydroxyglutarate (2-HG) levels in hematopoietic cells expressing mutant IDH2^R140Q^, and in murine models of IDH2-mutant leukemia. Consistent with its role in epigenetic dysregulation, mutant IDH2 inhibition with AG-221 reversed DNA hypermethylation in LSK stem cells from mice expressing mutant IDH2. Notably, AG-211 induced cell differentiation in leukemia cells from IDH2-mutant expressing mice and it synergized with Flt3 inhibition to reduce leukemic cell burden *in vivo*. AG-211 also demonstrated a survival benefit in primary human IDH2 mutant AML xenografts.^180, 181^ AG-211 has since been introduced into Phase I clinical trials and a Phase III trial has been initiated in 2015. Interim results presented thus far suggest that AG-211 is highly effective in decreasing plasma and bone marrow 2-HG levels and achieved durable remission in some patients with IDH2 mutant advanced hematologic malignancies.^182^ AGI-6780 is another selective inhibitor towards mutant IDH2^R140Q^.^183^ AGI-6780 treatment in IDH2 mutant cells resulted in histone and DNA demethylation,^184^ and reversed gene signatures caused by epigenetic dysregulation. These proof-of-concept studies indicate that targeting of mutant IDH1/IDH2 has potential clinical applications as a differentiation therapy in cancers bearing mutant forms of these proteins.

**SAM cycle inhibitors**

As the availability of SAM is critical for the activities of DNMTs and HMTs, SAM cycle blockade will likely affect DNA and histone methylation. *S*-adenosylhomocysteine hydrolase (SAH hydrolase) participates in the activated methyl cycle through catalyzing the hydrolysis of SAH into adenosine and homocysteine. SAH hydrolase is essential for the maintenance of methylation homeostasis, as SAH caused the byproduct inhibition of DNMTs and HMTs. DZNep (3-deazaneplanocin A) was first identified as a SAH hydrolase inhibitor,^185^ and subsequent studies showed that DZNep treatment in cancer cell lines globally inhibited DNA and histone methylation, an effect that is non-selective.^186^ Another SAH hydrolase inhibitor (adenosine dialdehyde) also had the similar impact on DNA and histone methylation. EZH2, an oncogenic HMT that methylates histone H3K27 and facilitates transcriptional repression, is indirectly targeted by DZNep in cancer cells via SAH hydrolase inhibition. DZNep treatment reactivates a subset of developmental genes, but it is ineffective towards genes silenced by dense promoter methylation. Hence, combination of DZNep with 5-aza-2′-deoxycytidine (5-Aza), a DNMT inhibitor, has shown synergistic anticancer activity in leukemia and colorectal cancer,^187, 188, 189, 190^ by activating genes that are aberrantly silenced by histone and DNA methylation.

**NNMT inhibitor**

As NNMT overexpression induced SAM depletion and histone hypomethylation, it might be a potential drug target in NNMT-overexpressing cancer cells. *N*-methyl-nicotinamide, a reaction side product of NNMT, is a specific and potent inhibitor towards NNMT. Indeed, NNMT inhibition *in vivo* using *N*-methylnicotinamide is able to increase histone methylation at H3K4 and increased methylated H3K4 occupancy at gene promoters.^191^ Thus, inhibition of NNMT is a viable approach for modification of histone methylation.

**Hexosamine biosynthesis pathway inhibitors**

UDP-GlcNAc, the substrate for protein *O*-GlcNAcylation, is synthesized via HBP. GFAT is the rate-limiting enzyme in HBP that can be targeted by well characterized inhibitors, *O*-diazoacetyl-L-serine (azaserine) and 6-diazo-5-oxo-L-norleucine (DON).^192^ Cancer cells cultured under high glucose exhibited increased HBP pathway flux and protein *O*-GlcNAcylation, which mediates transcriptional activation of β-catenin, thereby promoting the Wnt/β-catenin signaling and cell proliferation.^193, 194, 195^ Treatment with either azaserine or DON decreased protein *O*-GlcNAcylation level, reduced β-catenin expression and reversed glucose-mediated cell proliferation.^193, 194^ Protein *O*-GlcNAcylation has also been shown to be upregulated in CD133^+^ cancer stem cells.^196^ Inhibition of HBP using azaserine reduced the CD133^+^ subpopulation and CD133 expression; whereas treatment with *N*- *N*-acetylglucosamine (GlcNAc, which promotes HBP) had a reverse effect. It will be of great interest to investigate whether intervention of HBP will impact epigenetic regulators and histone modifications.

### Modulation of cancer metabolism using epigenetic drugs

#### DNMT inhibitors

DNA methylation can be therapeutically targeted using DNMT inhibitors. Two inhibitors of DNMT, 5-azacytidine and 5-aza-2′-deoxycytidine, have been clinically approved by FDA for treatment of myelodysplastic syndrome, and the latter has been approved for acute myeloid leukemia (AML). Clinical trials (Phase I–III) have also been conducted in several solid malignancies (Table 2). These are cytosine analogues that non-selectively inactivate DNMT1, DNMT3A and DNMT3B.^197^ It is largely unknown whether DNMT inhibitors can have a metabolic effect on cancer. Given the non-specific nature of these DNA methylation inhibitors and their widespread effect on gene expression, it will be important to elucidate their roles in cancer metabolism. DNMT inhibitors may be useful in reversing DNA methylation induced by metabolic alteration. IDH1/2-mutant cancers, which exhibit DNA hypermethylation, are sensitive to 5-azacytidine and 5-aza-2′-deoxycytidine. 5-Azacytidine induced tumor regression in a patient-derived IDH1 mutant glioma xenograft model;^198^ while 5-aza-2′-deoxycytidine effectively suppressed growth in IDH-mutant glioma cells *in vitro* and *in vivo*.^199^ DNMT inhibitors reversed the hypermethylator phenotype and resulted in cell differentiation and slowed growth. In the latter study, 5-aza-2′-deoxycytidine was actually shown to be more effective than IDH inhibitors in inducing the differentiation of IDH-mutant glioma cells. Hence, targeting the methylome may be a complementary approach to counteract the effect of oncometabolites in cancers.

#### HDAC inhibitors

HDAC inhibitors represent a diverse class of compounds that inhibit HDACs activity. Two HDAC inhibitors Vorinostat and Romidepsin have been approved for cutaneous T-cell lymphoma, and their use in solid tumors is an area of active investigation (Table 2). HDAC inhibitors induce histone acetylation and reverse gene silencing by HDACs in human cancers. Emerging evidence suggests that inhibition of HDACs may impact cancer metabolism. In HT29 colorectal cancer cells, treatment with HDAC inhibitors butyrate or trichostatin A was associated with a significant reduction in glucose uptake, glycolysis flux and lactate production.^200^ In multiple myeloma cells, Vorinostat or valproic acid treatment suppressed GLUT1 expression and inhibited hexokinase I activity.^201^ In H460 lung cancer cells, butyrate or trichostatin A treatment suppressed glycolysis and triggered a shift in metabolism away from glycolysis by activating mitochondrial metabolism.^202^ Butyrate also attenuated glycolysis in breast cancer cells.^203^ These results suggest that inhibition of HDAC may promote the reversion of glycolytic phenotype in cancer cells.

#### Sirtuin activators and inhibitors

Despite their importance in metabolic regulation in cancers, limited attention has been paid to the potential use of sirtuin activators and inhibitors to influence cancer metabolism. Sirtuin 6 (SIRT6), a tumor suppressor that opposes glycolysis, has been shown to be activated by free fatty acids (myristic, oleic and linoleic acids) up to ⩽35-fold.^204^ Discovery of small molecule activators of SIRT6 may unveil a novel approach to target tumor metabolism. On the other hand, several inhibitors that block oncogenic Sirtuin 2 (SIRT2) has been described.^205^ Further investigations are required to define the effect of sirtuin activators and inhibitors on cancer metabolism and their role in cancer management.

#### miRNA

Modulation of miRNAs holds promise as therapeutic targets, given its regulatory roles in the dysregulation of metabolism in carcinogenesis. Currently, there are two approaches to target miRNAs. Aberrantly silenced miRNAs can be restored using synthetic miRNA mimics; although overexpressed miRNAs can be silenced using miRNA sponges or antisense oligonucleotides.^206, 207, 208^ Both approaches have been utilized to manipulate metabolic genes in cancer. As an example, the re-expression of miR-143, which targets hexokinase II 3′-untranslated region, overturned the glycolytic phenotype and inhibited cancer growth.^154^ On the contrary, oncogenic miRNAs that target LKB1/AMPK or PTEN tumor suppressive pathways may represent attractive targets for the design of therapeutic anti-miRNAs. For instance, anti-miR-21-mediated inhibition (which targets PTEN tumor suppressor), restored PTEN expression in hepatocellular cancer and contributed to treatment.^209^ One major challenge facing miRNA-targeting focuses on the safe and efficient delivery of miRNA mimics and anti-miRNAs.^210^ Advances in delivery technology will accelerate realization of miRNA-based therapeutics in the clinical practice.

### Impact of tumor microenvironment and metabolism on epigenetic therapy

Epigenetic drugs, including DNMT and HDAC inhibitors, have been approved by the FDA for use in hematological malignancies. However, the use of these drugs has been met with limited success in solid tumors thus far. Several Phase I clinical trials that examined the pharmacodynamics of DNA demethylation drugs or histone deacetylase inhibitors indicated the reversal of epigenetic abnormalities in solid tumors following drug treatment.^211, 212, 213^ However, the therapeutic effects of epigenetic therapies towards solid tumors has been disappointing. Solid tumors, unlike hematological malignancies, are characterized by regions of hypoxia (low oxygen), which has a key role in tumor progression, aggressiveness and drug resistance.^214, 215^ Hypoxic tumor cells display epigenetic abnormalities.^216, 217, 218, 219, 220^ Hypoxia is associated with DNA hypomethylation. Hypoxia has been shown to downregulate the expression of DNMT1, DNMT3A and DNMT3B in human colorectal cancer cells.^217^ On the contrary, hypoxia results in HIF-dependent transcriptional activation of TET1.^219, 220^ Given that a hypoxic tumor microenvironment promotes DNA hypomethylation, efficacy of DNA demethylation agents, such as modulators of DNMTs/TETs, are likely suppressed in solid tumors as compared to hematological malignancies. On the other hand, hypoxia induces histone hypoacetylation that may be potentially targeted by inhibitors of HDAC.^221^ Indeed, HDAC inhibitors such as Vorinostat and valproic acid have shown promising results in solid tumors, especially given in combination with chemotherapeutic agents.^222, 223, 224, 225^ Therefore, approaches to target epigenetic mechanisms should take into consideration of the potential impact of the tumor microenvironment and metabolism.

## Conclusion and 5-year view

Crosstalks between epigenetics and metabolism are fundamental aspects of cellular adaptation to nutrition status. The human epigenome is dynamically regulated by the metabolome. Alterations in either the epigenome or metabolome arising from genetic mutation may therefore coordinately drive aberrant gene expression, which in turn, contributes to tumor development and progression. Here we have outlined potential strategies that target crosstalks between epigenome and metabolome that might be exploited to selectively inhibit tumorigenesis. At present, much of our knowledge has been gathered in simplistic *in vitro* cell culture systems that might poorly reflect the complex interaction of epigenome and metabolome *in vivo*. In the future, preclinical studies need to better define their crosstalk in context of the tumor microenvironment that consists of stromal and immune components and to validate potential targets using appropriate *in vivo* models, which ultimately will contribute to development of novel therapeutic targets for intervention.

With numerous drugs targeting metabolism in the drug development pipeline, in the next 5 years we will be able to effectively target these abnormalities in cancer. Novel drugs targeting mutant IDH1/2, for example, are already undergoing phase II/III trials with treatment of advanced leukemia harboring these mutations. With the promising preliminary data, IDH1/2 inhibition represents a highly specific therapy for this subset of cancers.^183, 184^ Metabolic reprogramming in cancer cells might also be targeted by epigenetic drugs such as DNMT and HDAC inhibitors. However, targeting epigenetic machinery likely has a broad impact on gene expression, and more studies are needed to define their specific effects on tumor metabolism. A caveat of targeted therapies, as exemplified by the development of tyrosine kinase inhibitors, is that they are useful only when their target(s) are the main drivers of carcinogenesis. To fully realize the potential of metabolic/epigenetic modulators, future clinical trials should incorporate analysis of biomarkers to unravel epigenomic (DNA methylation and histone lysine acetylation) and metabolomic (metabolites) markers that allow the selection of subsets of patients that may benefit most from these treatments. Given the extensive crosstalk between epigenetics and metabolism, perhaps it is the development of combinatorial approaches involving metabolism inhibitors and epigenetic modulators might achieve synergistic tumor inhibition. Notably, mutant IDH1/2 inhibitors are being evaluated in Phase I/II clinical trials with inhibitors of DNMTs (Table 1), with the rationale being that they might promote active DNA demethylation and suppress DNA methylation, respectively, to reverse DNA methylation in IDH1/2-mutant cancers. Development of rationale drug combinations involving metabolism inhibitors, epigenetic modulators and traditional chemotherapeutics will likely have the greatest impact on future cancer management.

## Figures and Tables

**Table 1:** Reversal of epigenetic dysregulation by targeting cancer metabolism

*Inhibitor*	*Target enzyme*	*Mode of action*	*Ongoing clinical use/trials*	*Ref*
*Glycolysis inhibitors*				
2-Deoxyglucose (2-DG)	Hexokinases	2-DG inhibits hexokinase, a rate limiting enzyme for glycolysis. 2-DG suppressed acetyl-CoA levels, and reduced the acetylation of histone H3, H4, H2A and H2B in multiple cancer cell lines	2-Deoxyglucose (Phase I/II)	^164, 165^
3-Bromopyruvate (BrPA)	Glyceraldehyde 3-phosphate dehydrogenase	BrPA inhibits GAPDH and acetyl-CoA production BrPA suppressed histone acetylation and induced differentiation of embryonic stem cells	NA	^166^

*Glutaminolysis inhibitors*				
Bis-2-(5-phenylacetamido-1,2,4-thiadiazol-2-yl) ethyl sulfide (BPTES), CB-839, Compound 968, Zaprinast	Glutaminase (GLS)	GLS inhibitors suppress acetyl CoA and 2-HG production Compound 968 reduced histone H3K4me3 in breast cancer cells. Zaprinast decreased H3K9me2/3 in IDH1-mutant cancer cells	CB-839 (Phase I)	^167, 168, 169, 170, 171^

*IDH1 inhibitors*				
AG-120, AG-881, AGI-5198, BAY1436032, bis imidazole phenol, FT-2102, GSK321, GSK864, 1-Hydroxypyridin-2-one compounds, IDH305, ML309, 2-(3-Trifluoromethylphenyl)isothioazol-3(2H)-one	Mutant IDH1	IDH1 inhibitors suppressed 2-HG production in IDH1 mutant cells. AGI-5198 induced demethylation of H3K9me3 and H3K27me3 in IDH1-mutant chondrosarcoma cells. GSK321 induced genome-wide DNA hypomethylation in IDH1 mutant acute myeloid leukemia cells	AG-120±Azacitidine (Phase I/II), AG-881 (Phase I), BAY1436032 (Phase I), FT-2102±Azacitidine (Phase I), IDH305 (Phase I)	^172, 173, 174, 175, 176, 177, 178^

*IDH2 inhibitors*				
AG-221, AG-881, AGI-6780	Mutant IDH2	IDH2 inhibitors suppressed 2-HG production in IDH2-mutant cells. Both AG-221 and AGI-6780 induced demethylation of DNA and histone marks in IDH2-mutant leukemia cell lines	AG-221±Azacitidine (Phase I/III), AG-881 (Phase I)	^179, 180, 181, 182, 183, 184^

*SAM cycle inhibitors*				
DZNep (3-deazaneplanocin A), adenosine dialdehyde	SAH hydrolase	DZNep and adenosine dialdehyde increased the SAH-to-SAM ratio and inhibited DNA and histone methylation in cancer cell lines	NA	^185, 186, 187, 188, 189, 190^

*NNMT inhibitor*				
*N*-Methylnicotinamide	Nicotinamide *N*-methyl-transferase (NNMT)	*N*-methylnicotinamide increased SAM level and histone methylation in NNMT-overexpressing cancer cells	NA	^191^

*Hexosamine biosynthesis pathway inhibitors*				
*O*-Diazoacetyl-L-serine (azaserine), 6-Diazo-5-oxo-L-norleucine (DON)	Glutamine-Fructose-6-Phosphate Transaminase	Azaserine and DON decreased protein *O*-GlcNAcylation levels	NA	^192^

Abbreviation: NA, not applicable.

**Table 2:** Reversal of cancer metabolism using epigenetic drugs

*Inhibitor*	*Target enzyme*	*Mode of Action*	*Ongoing clinical use/trials*	*Ref*
*DNMT inhibitors*				
5-Azacytidine, 5-Aza-2'-deoxycytidine	DNA methyltransferases	Both drugs non-selectively inactivate DNMT1, DNMT3A and DNMT3B DNMT inhibitors reversed the hypermethylator phenotype in IDH1-mutant glioma cells	Azacitidine and 5-Aza-2′-deoxycytidine (Approved for myelodysplastic syndrome and acute myeloid leukemia, Phase I-III for other malignancies)	^198, 199^

*HDAC inhibitors*				
Butyrate, Romidepsin, Trichostatin A, Valproic acid, Vorinostat	Histone deacetylases (HDACs)	HDAC inhibitors induce histone acetylation and reverse aberrant gene expression caused by HDACs. Treatment of cancer cells with HDAC inhibitors was associated with the reduction in glucose uptake, glycolytic flux and lactate metabolism	Romidepsin and Vorinostat (Approved for cutaneous T cell lymphoma, Phase I-III for other malignancies), Valproic acid (Phase I-III)	^200, 201, 202, 203^

*Sirtuin activators and inhibitors*				
Linoleic acid, Myristic acid, Oleic acid	Sirtuin 6 (SIRT6)	Free fatty acids activate SIRT6, which functions as a tumor suppressor to inhibit glycolysis	NA	^204^

*miRNA modulators*				
Synthetic miRNA mimics	miRNAs	miRNA mimics restores silenced miRNA function. For example, re-expression of miR-143, which targets hexokinase II 3′-UTR, suppressed glycolysis	NA	^154^
miRNA sponges, Antisense oligonucleotides	miRNAs	Anti-miRs silences overexpressed miRNA. For example, anti-miR-21 restored PTEN expression	NA	^209^

Abbreviations: HDAC, histone deacetylase; miRNA, microRNA; NA, not applicable; 3′-UTR, 3′-untranslated region.

## References

[bib1] Warburg O. On the origin of cancer cells. Science 1956; 123: 309–314. 13298683 10.1126/science.123.3191.309

[bib2] Kim JW, Dang CV. Cancer's molecular sweet tooth and the Warburg effect. Cancer Res 2006; 66: 8927–8930. 16982728 10.1158/0008-5472.CAN-06-1501

[bib3] Vander Heiden MG, Cantley LC, Thompson CB. Understanding the Warburg effect: the metabolic requirements of cell proliferation. Science 2009; 324: 1029–1033. 19460998 10.1126/science.1160809 PMC2849637

[bib4] Dang CV. Glutaminolysis: supplying carbon or nitrogen or both for cancer cells? Cell Cycle 2010; 9: 3884–3886. 20948290 10.4161/cc.9.19.13302

[bib5] Jin L, Alesi GN, Kang S. Glutaminolysis as a target for cancer therapy. Oncogene 2015; 35: 3619–3625. 26592449 10.1038/onc.2015.447 PMC5225500

[bib6] Wise DR, DeBerardinis RJ, Mancuso A, Sayed N, Zhang XY, Pfeiffer HK et al. Myc regulates a transcriptional program that stimulates mitochondrial glutaminolysis and leads to glutamine addiction. Proc Natl Acad Sci USA 2008; 105: 18782–18787. 19033189 10.1073/pnas.0810199105 PMC2596212

[bib7] Hensley CT, Wasti AT, DeBerardinis RJ. Glutamine and cancer: cell biology, physiology, and clinical opportunities. J Clin Invest 2013; 123: 3678–3684. 23999442 10.1172/JCI69600 PMC3754270

[bib8] Wong CC, Qian Y, Li X, Xu J, Kang W, Tong JH et al. SLC25A22 promotes tumorigenicity and metastasis of KRAS-mutant colorectal cancer by regulating intracellular aspartate biosynthesis. Gastroenterology 2016; 151: 945–960. 27451147 10.1053/j.gastro.2016.07.011

[bib9] Barthel A, Okino ST, Liao J, Nakatani K, Li J, Whitlock JP Jr et al. Regulation of GLUT1 gene transcription by the serine/threonine kinase Akt1. J Biol Chem 1999; 274: 20281–20286. 10400647 10.1074/jbc.274.29.20281

[bib10] Yun J, Rago C, Cheong I, Pagliarini R, Angenendt P, Rajagopalan H et al. Glucose deprivation contributes to the development of KRAS pathway mutations in tumor cells. Science 2009; 325: 1555–1559. 19661383 10.1126/science.1174229 PMC2820374

[bib11] Morani F, Phadngam S, Follo C, Titone R, Aimaretti G, Galetto A et al. PTEN regulates plasma membrane expression of glucose transporter 1 and glucose uptake in thyroid cancer cells. J Mol Endocrinol 2014; 53: 247–258. 25125078 10.1530/JME-14-0118

[bib12] Miyamoto S, Murphy AN, Brown JH. Akt mediates mitochondrial protection in cardiomyocytes through phosphorylation of mitochondrial hexokinase-II. Cell Death Differ 2008; 15: 521–529. 18064042 10.1038/sj.cdd.4402285

[bib13] Deprez J, Vertommen D, Alessi DR, Hue L, Rider MH. Phosphorylation and activation of heart 6-phosphofructo-2-kinase by protein kinase B and other protein kinases of the insulin signaling cascades. J Biol Chem 1997; 272: 17269–17275. 9211863 10.1074/jbc.272.28.17269

[bib14] Osthus RC, Shim H, Kim S, Li Q, Reddy R, Mukherjee M et al. Deregulation of glucose transporter 1 and glycolytic gene expression by c-Myc. J Biol Chem 2000; 275: 21797–21800. 10823814 10.1074/jbc.C000023200

[bib15] David CJ, Chen M, Assanah M, Canoll P, Manley JL. HnRNP proteins controlled by c-Myc deregulate pyruvate kinase mRNA splicing in cancer. Nature 2010; 463: 364–368. 20010808 10.1038/nature08697 PMC2950088

[bib16] Yan H, Parsons W, Jin G, McLendon R, Rasheed A, Yuan W et al. IDH1 and IDH2 mutations in gliomas. N Engl J Med 2009; 360: 765–773. 19228619 10.1056/NEJMoa0808710 PMC2820383

[bib17] Figueroa ME, Abdel-Wahab O, Lu C, Ward PS, Patel J, Shih A et al. Leukemic IDH1 and IDH2 mutations result in a hypermethylation phenotype, disrupt TET2 function, and impair hematopoietic differentiation. Cancer Cell 2010; 18: 553–567. 21130701 10.1016/j.ccr.2010.11.015 PMC4105845

[bib18] Astuti D, Latif F, Dallol A, Dahia PL, Douglas F, George E et al. Gene mutations in the succinate dehydrogenase subunit SDHB cause susceptibility to familial pheochromocytoma and to familial paraganglioma. Am J Hum Genet 2001; 69: 49–54. 11404820 10.1086/321282 PMC1226047

[bib19] Toro JR, Nickerson ML, Wei MH, Warren MB, Glenn GM, Turner ML et al. Mutations in the fumarate hydratase gene cause hereditary leiomyomatosis and renal cell cancer in families in North America. Am J Hum Genet 2003; 73: 95–106. 12772087 10.1086/376435 PMC1180594

[bib20] Hanahan D, Weinberg RA. Hallmarks of cancer: the next generation. Cell 2011; 144: 646–674. 21376230 10.1016/j.cell.2011.02.013

[bib21] Jaenisch R, Bird A. Epigenetic regulation of gene expression: how the genome integrates intrinsic and environmental signals. Nat Genet 2003; 33S: 245–254. 10.1038/ng1089 12610534

[bib22] Feil R, Fraga MF. Epigenetics and the environment: emerging patterns and implications. Nat Rev Genet 2011; 13: 97–109. 10.1038/nrg3142 22215131

[bib23] Herceg Z, Vaissiere T. Epigenetic mechanisms and cancer: an interface between the environment and the genome. Epigenetics 2011; 6: 804–819. 21758002 10.4161/epi.6.7.16262

[bib24] Gupta V, Gopinath P, Iqbal MA, Mazurek S, Wellen KE, Bamezai RN. Interplay between epigenetics & cancer metabolism. Curr Pharm Des 2014; 20: 1706–1714. 23888952 10.2174/13816128113199990536

[bib25] Johnson C, Warmoes MO, Shen X, Locasale JW. Epigenetics and cancer metabolism. Cancer Lett 2015; 356: 309–314. 24125862 10.1016/j.canlet.2013.09.043 PMC3984372

[bib26] Munoz-Pinedo C, Gonzalez-Suarez E, Portela A, Gentilella A, Esteller M. Exploiting tumor vulnerabilities: epigenetics, cancer metabolism and the mTOR pathway in the era of personalized medicine. Cancer Res 2013; 73: 4185–4189. 23687347 10.1158/0008-5472.CAN-13-0512

[bib27] Watt F, Molloy PL. Cytosine methylation prevents binding to DNA of a HeLa cell transcription factor required for optimal expression of the adenovirus major late promoter. Genes Dev 1988; 2: 1136–1143. 3192075 10.1101/gad.2.9.1136

[bib28] Ng HH, Zhang Y, Hendrich B, Johnson CA, Turner BM, Erdjument-Bromage H et al. MBD2 is a transcriptional repressor belonging to the MeCP1 histone deacetylase complex. Nat Genet 1999; 23: 58–61. 10471499 10.1038/12659

[bib29] Selvakumar T, Gjidoda A, Hovde SL, Henry RW. Regulation of human RNA polymerase III transcription by DNMT1 and DNMT3a DNA methyltransferases. J Biol Chem 2012; 287: 7039–7050. 22219193 10.1074/jbc.M111.285601 PMC3293528

[bib30] Ying H, Kimmelman AC, Lyssiotis CA, Hua S, Chu GC, Fletcher-Sananikone E et al. Oncogenic Kras maintains pancreatic tumors through regulation of anabolic glucose metabolism. Cell 2012; 149: 656–670. 22541435 10.1016/j.cell.2012.01.058 PMC3472002

[bib31] Liu W, Li X, Chu ES, Go MY, Xu L, Zhao G et al. Paired box gene 5 is a novel tumor suppressor in hepatocellular carcinoma through interaction with p53 signaling pathway. Hepatology 2011; 53: 843–853. 21319196 10.1002/hep.24124

[bib32] Wang S, Cheng Y, Du W, Lu L, Zhou L, Wang H et al. Zinc-finger protein 545 is a novel tumour suppressor that acts by inhibiting ribosomal RNA transcription in gastric cancer. Gut 2013; 62: 833–841. 22580414 10.1136/gutjnl-2011-301776

[bib33] Yu J, Liang QY, Wang J, Cheng Y, Wang S, Poon TC et al. Zinc-finger protein 331, a novel putative tumor suppressor, suppresses growth and invasiveness of gastric cancer. Oncogene 2013; 32: 307–317. 22370639 10.1038/onc.2012.54

[bib34] Shen L, Kondo Y, Rosner GL, Xiao L, Hernandez NS, Vilaythong J et al. MGMT promoter methylation and field defect in sporadic colorectal cancer. J Natl Cancer Inst 2005; 97: 1330–1338. 16174854 10.1093/jnci/dji275

[bib35] Du W, Wang S, Zhou Q, Li X, Chu J, Chang Z et al. ADAMTS9 is a functional tumor suppressor through inhibiting AKT/mTOR pathway and associated with poor survival in gastric cancer. Oncogene 2013; 32: 3319–3328. 22907434 10.1038/onc.2012.359

[bib36] Choi GC, Li J, Wang Y, Li L, Zhong L, Ma B et al. The metalloprotease ADAMTS8 displays antitumor properties through antagonizing EGFR-MEK- ERK signaling and is silenced in carcinomas by CpG methylation. Mol Cancer Res 2014; 12: 228–238. 24184540 10.1158/1541-7786.MCR-13-0195

[bib37] Patel SA, Graunke DM, Pieper RO. Aberrant silencing of the CpG island-containing human O6-methylguanine DNA methyltransferase gene is associated with the loss of nucleosome-like positioning. Mol Cell Biol 1997; 17: 5813–5822. 9315639 10.1128/mcb.17.10.5813 PMC232429

[bib38] Cunningham JM, Christensen ER, Tester DJ, Kim CY, Roche PC, Burgart LJ et al. Hypermethylation of the hMLH1 promoter in colon cancer with microsatellite instability. Cancer Res 1998; 58: 3455–3460. 9699680

[bib39] Robertson KD, Uzvolgyi E, Liang G, Talmadge C, Sumegi J, Gonzales FA et al. The human DNA methyltransferases (DNMTs) 1, 3a and 3b: coordinate mRNA expression in normal tissues and overexpression in tumors. Nucleic Acids Res 1999; 27: 2291–2298. 10325416 10.1093/nar/27.11.2291 PMC148793

[bib40] Zhang Y, Reinberg D. Transcription regulation by histone methylation: interplay between different covalent modifications of the core histone tails. Genes Dev 2001; 15: 2343–2360. 11562345 10.1101/gad.927301

[bib41] Greer EL, Shi Y. Histone methylation: a dynamic mark in health, disease and inheritance. Nat Rev Genet 2012; 13: 343–357. 22473383 10.1038/nrg3173 PMC4073795

[bib42] Mentch SJ, Mehrmohamadi M, Huang L, Liu X, Gupta D, Mattocks D et al. Histone Methylation Dynamics and Gene Regulation Occur through the Sensing of One-Carbon Metabolism. Cell Metab 2015; 22: 861–873. 26411344 10.1016/j.cmet.2015.08.024 PMC4635069

[bib43] Mudd SH, Cerone R, Schiaffino MC, Fantasia AR, Minniti G, Caruso U et al. Glycine N-methyltransferase deficiency: a novel inborn error causing persistent isolated hypermethioninaemia. J Inherit Metab Dis 2001; 24: 448–464. 11596649 10.1023/a:1010577512912

[bib44] Martínez-Chantar ML, Vázquez-Chantada M, Ariz U, Martínez N, Varela M, Luka Z et al. Loss of the glycine N-methyltransferase gene leads to steatosis and hepatocellular carcinoma in mice. Hepatology 2008; 47: 1191–1199. 18318442 10.1002/hep.22159 PMC2405897

[bib45] Fuchs BC, Bode BP. Amino acid transporters ASCT2 and LAT1 in cancer: partners in crime? Semin Cancer Biol 2005; 15: 254–266. 15916903 10.1016/j.semcancer.2005.04.005

[bib46] Haase C, Bergmann R, Fuechtner F, Hoepping A, Pietzsch J. L-type amino acid transporters LAT1 and LAT4 in cancer: uptake of 3-O-methyl-6-18 F-fluoro-L-dopa in human adenocarcinoma and squamous cell carcinoma*in vitro*and*in vivo*. J Nucl Med 2007; 48: 2063–2071. 18056335 10.2967/jnumed.107.043620

[bib47] Possemato R, Marks KM, Shaul YD, Pacold ME, Kim D, Birsoy K et al. Functional genomics reveal that the serine synthesis pathway is essential in breast cancer. Nature 2011; 476: 346–350. 21760589 10.1038/nature10350 PMC3353325

[bib48] Locasale JW, Grassian AR, Melman T, Lyssiotis CA, Mattaini KR, Bass AJ et al. Phosphoglycerate dehydrogenase diverts glycolytic flux and contributes to oncogenesis. Nat Genet 2011; 43: 869–874. 21804546 10.1038/ng.890 PMC3677549

[bib49] Maddocks OD, Labuschagne CF, Adams PD, Vousden KH. Serine metabolism supports the methionine cycle and DNA/RNA methylation through de novo ATP synthesis in cancer cells. Mol Cell 2016; 61: 210–221. 26774282 10.1016/j.molcel.2015.12.014 PMC4728077

[bib50] Wu Y, Siadaty MS, Berens ME, Hampton GM, Theodorescu D. Overlapping gene expression profiles of cell migration and tumor invasion in human bladder cancer identify metallothionein 1E and nicotinamide N-methyltransferase as novel regulators of cell migration. Oncogene 2008; 27: 6679–6689. 18724390 10.1038/onc.2008.264 PMC5373842

[bib51] Zhang J, Wang Y, Li G, Yu H, Xie X. Downregulation of nicotinamide N-methyltransferase induces apoptosis in human breast cancer cells via the mitochondria-mediated pathway. PLoS One 2014; 9: e89202. 24558488 10.1371/journal.pone.0089202 PMC3928407

[bib52] Thomas MG, Saldanha M, Mistry RJ, Dexter DT, Ramsden DB, Parsons RB et al. Nicotinamide N-methyltransferase expression in SH-SY5Y neuroblastoma and N27 mesencephalic neurones induces changes in cell morphology via ephrin-B2 and Akt signalling. Cell Death Dis 2013; 4: e669. 23764850 10.1038/cddis.2013.200 PMC3702289

[bib53] Tang SW, Yang TC, Lin WC, Chang WH, Wang CC, Lai MK et al. Nicotinamide N-methyltransferase induces cellular invasion through activating matrix metalloproteinase-2 expression in clear cell renal cell carcinoma cells. Carcinogenesis 2011; 32: 138–145. 21045016 10.1093/carcin/bgq225

[bib54] Ulanovskaya OA, Zuhl AM, Cravatt BF. NNMT promotes epigenetic remodeling in cancer by creating a metabolic methylation sink. Nat Chem Biol 2013; 9: 300–306. 23455543 10.1038/nchembio.1204 PMC3631284

[bib55] Guo JU, Su Y, Zhong C, Ming GL, Song H. Hydroxylation of 5-methylcytosine by TET1 promotes active DNA demethylation in the adult brain. Cell 2011; 145: 423–434. 21496894 10.1016/j.cell.2011.03.022 PMC3088758

[bib56] Tahiliani M, Koh KP, Shen Y, Pastor WA, Bandukwala H, Brudno Y et al. Conversion of 5-methylcytosine to 5-hydroxymethylcytosine in mammalian DNA by MLL partner TET1. Science 2009; 324: 930–935. 19372391 10.1126/science.1170116 PMC2715015

[bib57] Ito S, D'Alessio AC, Taranova OV, Hong K, Sowers LC, Zhang Y. Role of Tet proteins in 5mC to 5hmC conversion, ES-cell self-renewal and inner cell mass specification. Nature 2010; 466: 1129–1133. 20639862 10.1038/nature09303 PMC3491567

[bib58] Kohli RM, Zhang Y. TET enzymes, TDG and the dynamics of DNA demethylation. Nature 2013; 502: 472–479. 24153300 10.1038/nature12750 PMC4046508

[bib59] Abdel-Wahab O, Mullally A, Hedvat C, Garcia-Manero G, Patel J, Wadleigh M et al. Genetic characterization of TET1, TET2, and TET3 alterations in myeloid malignancies. Blood 2009; 114: 144–147. 19420352 10.1182/blood-2009-03-210039 PMC2710942

[bib60] Gambichler T, Sand M, Skrygan M. Loss of 5-hydroxymethylcytosine and ten-eleven translocation 2 protein expression in malignant melanoma. Melanoma Res 2013; 23: 218–220. 23458759 10.1097/CMR.0b013e32835f9bd4

[bib61] Yang H, Liu Y, Bai F, Zhang JY, Ma SH, Liu J et al. Tumor development is associated with decrease of TET gene expression and 5-methylcytosine hydroxylation. Oncogene 2013; 32: 663–669. 22391558 10.1038/onc.2012.67 PMC3897214

[bib62] Kudo Y, Tateishi K, Yamamoto K, Yamamoto S, Asaoka Y, Ijichi H et al. Loss of 5-hydroxymethylcytosine is accompanied with malignant cellular transformation. Cancer Sci 2012; 103: 670–676. 22320381 10.1111/j.1349-7006.2012.02213.x PMC7659252

[bib63] Kooistra SM, Helin K. Molecular mechanisms and potential functions of histone demethylases. Nat Rev Mol Cell Biol 2012; 13: 297–311. 22473470 10.1038/nrm3327

[bib64] Wu J, Liu S, Liu G, Dombkowski A, Abrams J, Martin-Trevino R et al. Identification and functional analysis of 9p24 amplified genes in human breast cancer. Oncogene 2012; 31: 333–341. 21666724 10.1038/onc.2011.227 PMC3886828

[bib65] Schulte JH, Lim S, Schramm A, Friedrichs N, Koster J, Versteeg R et al. Lysine-specific demethylase 1 is strongly expressed in poorly differentiated neuroblastoma: implications for therapy. Cancer Res 2009; 69: 2065–2071. 19223552 10.1158/0008-5472.CAN-08-1735

[bib66] Xiao M, Yang H, Xu W, Ma S, Lin H, Zhu H et al. Inhibition of alpha-KG-dependent histone and DNA demethylases by fumarate and succinate that are accumulated in mutations of FH and SDH tumor suppressors. Genes Dev 2012; 26: 1326–1338. 22677546 10.1101/gad.191056.112 PMC3387660

[bib67] Balss J, Meyer J, Mueller W, Korshunov A, Hartmann C, von Deimling A. Analysis of the IDH1 codon 132 mutation in brain tumors. Acta Neuropathol 2008; 116: 597–602. 18985363 10.1007/s00401-008-0455-2

[bib68] Paschka P, Schlenk RF, Gaidzik VI, Habdank M, Krönke J, Bullinger L et al. IDH1 and IDH2 mutations are frequent genetic alterations in acute myeloid leukemia and confer adverse prognosis in cytogenetically normal acute myeloid leukemia with NPM1 mutation without FLT3 internal tandem duplication. J Clin Oncol 2010; 28: 3636–3643. 20567020 10.1200/JCO.2010.28.3762

[bib69] Ward PS, Patel J, Wise DR, Abdel-Wahab O, Bennett BD, Coller HA et al. The common feature of leukemia-associated IDH1 and IDH2 mutations is a neomorphic enzyme activity converting alpha-ketoglutarate to 2-hydroxyglutarate. Cancer Cell 2010; 17: 225–234. 20171147 10.1016/j.ccr.2010.01.020 PMC2849316

[bib70] Cairns RA, Iqbal J, Lemonnier F, Kucuk C, de Leval L, Jais JP et al. IDH2 mutations are frequent in angioimmunoblastic T-cell lymphoma. Blood 2012; 119: 1901–1903. 22215888 10.1182/blood-2011-11-391748 PMC3293643

[bib71] Hurley JH, Dean AM, Koshland DE, Stroud RM. Catalytic mechanism of NADP(+)-dependent isocitrate dehydrogenase: implications from the structures of magnesium-isocitrate and NADP+ complexes. Biochemistry 1991; 30: 8671–8678. 1888729 10.1021/bi00099a026

[bib72] Dang L, White DW, Gross S, Bennett BD, Bittinger MA, Driggers EM et al. Cancer-associated IDH1 mutations produce 2-hydroxyglutarate. Nature 2009; 462: 739–744. 19935646 10.1038/nature08617 PMC2818760

[bib73] Lu C, Ward PS, Kapoor GS, Rohle D, Turcan S, Abdel-Wahab O et al. IDH mutation impairs histone demethylation and results in a block to cell differentiation. Nature 2012; 483: 474–478. 22343901 10.1038/nature10860 PMC3478770

[bib74] Sasaki M, Knobbe CB, Munger JC, Lind EF, Brenner D, Brüstle A et al. IDH1(R132H) mutation increases murine haematopoietic progenitors and alters epigenetics. Nature 2012; 488: 656–659. 22763442 10.1038/nature11323 PMC4005896

[bib75] Terunuma A, Putluri N, Mishra P, Mathé EA, Dorsey TH, Yi M et al. MYC-driven accumulation of 2-hydroxyglutarate is associated with breast cancer prognosis. J Clin Invest 2014; 124: 398–412. 24316975 10.1172/JCI71180 PMC3871244

[bib76] Shim EH, Livi CB, Rakheja D, Tan J, Benson D, Parekh V et al. L-2-hydroxyglutarate: an epigenetic modifier and putative oncometabolite in renal cancer. Cancer Discov 2014; 4: 1290–1298. 25182153 10.1158/2159-8290.CD-13-0696 PMC4286872

[bib77] Rzem R, Veiga-da-Cunha M, Noël G, Goffette S, Nassogne MC, Tabarki B et al. A gene encoding a putative FAD-dependent L-2-hydroxyglutarate dehydrogenase is mutated in L-2-hydroxyglutaric aciduria. Proc Natl Acad Sci USAUSA 2004; 101: 16849–16854. 10.1073/pnas.0404840101 PMC534725 15548604

[bib78] Chowdhury R, Yeoh KK, Tian YM, Hillringhaus L, Bagg EA, Rose NR et al. The oncometabolite 2-hydroxyglutarate inhibits histone lysine demethylases. EMBO Rep 2011; 12: 463–469. 21460794 10.1038/embor.2011.43 PMC3090014

[bib79] Xu W, Yang H, Liu Y, Yang Y, Wang P, Kim SH et al. Oncometabolite 2-hydroxyglutarate is a competitive inhibitor of alpha-ketoglutarate-dependent dioxygenases. Cancer Cell 2011; 19: 17–30. 21251613 10.1016/j.ccr.2010.12.014 PMC3229304

[bib80] Christensen BC, Smith AA, Zheng S, Koestler DC, Houseman EA, Marsit CJ et al. DNA methylation, isocitrate dehydrogenase mutation, and survival in glioma. J Natl Cancer Inst 2011; 103: 143–153. 21163902 10.1093/jnci/djq497 PMC3022619

[bib81] Turcan S, Rohle D, Goenka A, Walsh LA, Fang F, Yilmaz E et al. IDH1 mutation is sufficient to establish the glioma hypermethylator phenotype. Nature 2012; 483: 479–483. 22343889 10.1038/nature10866 PMC3351699

[bib82] Venneti S, Felicella MM, Coyne T, Phillips JJ, Gorovets D, Huse JT et al. Histone 3 lysine 9 trimethylation is differentially associated with isocitrate dehydrogenase mutations in oligodendrogliomas and high-grade astrocytomas. J Neuropathol Exp Neurol 2013; 72: 298–306. 23481705 10.1097/NEN.0b013e3182898113 PMC3615673

[bib83] Pollard PJ, Wortham NC, Tomlinson IP. The TCA cycle and tumorigenesis: the examples of fumarate hydratase and succinate dehydrogenase. Ann Med 2003; 35: 632–639. 14708972 10.1080/07853890310018458

[bib84] Janeway KA, Kim SY, Lodish M, Nosé V, Rustin P, Gaal J et al. Defects in succinate dehydrogenase in gastrointestinal stromal tumors lacking KIT and PDGFRA mutations. Proc Natl Acad Sci USA 2011; 108: 314–318. 21173220 10.1073/pnas.1009199108 PMC3017134

[bib85] Ricketts CJ, Shuch B, Vocke CD, Metwalli AR, Bratslavsky G, Middelton L et al. Succinate dehydrogenase kidney cancer: an aggressive example of the Warburg effect in cancer. J Urol 2012; 188: 2063–2071. 23083876 10.1016/j.juro.2012.08.030 PMC3856891

[bib86] Hensen EF, Bayley JP. Recent advances in the genetics of SDH-related paraganglioma and pheochromocytoma. Fam Cancer 2011; 10: 355–363. 21082267 10.1007/s10689-010-9402-1 PMC3100491

[bib87] Kantorovich V, King KS, Pacak K. SDH-related pheochromocytoma and paraganglioma. Best Pract Res Clin Endocrinol Metab 2010; 24: 415–424. 20833333 10.1016/j.beem.2010.04.001 PMC2939070

[bib88] Hao HX, Khalimonchuk O, Schraders M, Dephoure N, Bayley JP, Kunst H et al. SDH5, a gene required for flavination of succinate dehydrogenase, is mutated in paraganglioma. Science 2009; 325: 1139–1142. 19628817 10.1126/science.1175689 PMC3881419

[bib89] Letouzé E, Martinelli C, Loriot C, Burnichon N, Abermil N, Ottolenghi C et al. SDH mutations establish a hypermethylator phenotype in paraganglioma. Cancer cell 2013; 23: 739–752. 23707781 10.1016/j.ccr.2013.04.018

[bib90] Killian JK, Kim SY, Miettinen M, Smith C, Merino M, Tsokos M et al. Succinate dehydrogenase mutation underlies global epigenomic divergence in gastrointestinal stromal tumor. Cancer Discov 2013; 3: 648–657. 23550148 10.1158/2159-8290.CD-13-0092 PMC4135374

[bib91] Eberharter A, Becker PB. Histone acetylation: a switch between repressive and permissive chromatin. Second in review series on chromatin dynamics. EMBO Rep 2002; 3: 224–229. 11882541 10.1093/embo-reports/kvf053 PMC1084017

[bib92] Nakagawa M, Oda Y, Eguchi T, Aishima S, Yao T, Hosoi F et al. Expression profile of class I histone deacetylases in human cancer tissues. Oncol Rep 2007; 18: 769–774. 17786334

[bib93] Weichert W, Röske A, Gekeler V, Beckers T, Stephan C, Jung K et al. Histone deacetylases 1, 2 and 3 are highly expressed in prostate cancer and HDAC2 expression is associated with shorter PSA relapse time after radical prostatectomy. Br J Cancer 2008; 98: 604–610. 18212746 10.1038/sj.bjc.6604199 PMC2243142

[bib94] Iyer NG, Ozdag H, Caldas C. p300/CBP and cancer. Oncogene 2004; 23: 4225–4231. 15156177 10.1038/sj.onc.1207118

[bib95] Gayther SA, Batley SJ, Linger L, Bannister A, Thorpe K, Chin SF et al. Mutations truncating the EP300 acetylase in human cancers. Nat Genet 2000; 24: 300–303. 10700188 10.1038/73536

[bib96] Wang J, Iwasaki H, Krivtsov A, Febbo PG, Thorner AR, Ernst P et al. Conditional MLL-CBP targets GMP and models therapy-related myeloproliferative disease. EMBO J 2005; 24: 368–381. 15635450 10.1038/sj.emboj.7600521 PMC545811

[bib97] Huntly BJ, Shigematsu H, Deguchi K, Lee BH, Mizuno S, Duclos N et al. MOZ-TIF2, but not BCR-ABL, confers properties of leukemic stem cells to committed murine hematopoietic progenitors. Cancer Cell 2004; 6: 587–596. 15607963 10.1016/j.ccr.2004.10.015

[bib98] Zaidi N, Swinnen JV, Smans K. ATP-citrate lyase: a key player in cancer metabolism. Cancer Res 2012; 72: 3709–3714. 22787121 10.1158/0008-5472.CAN-11-4112

[bib99] Lee JV, Carrer A, Shah S, Snyder NW, Wei S, Venneti S et al. Akt-dependent metabolic reprogramming regulates tumor cell histone acetylation. Cell Metab 2014; 20: 306–319. 24998913 10.1016/j.cmet.2014.06.004 PMC4151270

[bib100] Wellen KE, Hatzivassiliou G, Sachdeva UM, Bui TV, Cross JR, Thompson CB. ATP-citrate lyase links cellular metabolism to histone acetylation. Science 2009; 324: 1076–1080. 19461003 10.1126/science.1164097 PMC2746744

[bib101] Migita T, Narita T, Nomura K, Miyagi E, Inazuka F, Matsuura M et al. ATP citrate lyase: activation and therapeutic implications in non-small cell lung cancer. Cancer Res 2008; 68: 8547–8554. 18922930 10.1158/0008-5472.CAN-08-1235

[bib102] Knoepfler PS, Zhang XY, Cheng PF, Gafken PR, McMahon SB, Eisenman RN. Myc influences global chromatin structure. EMBO J 2006; 25: 2723–2734. 16724113 10.1038/sj.emboj.7601152 PMC1500848

[bib103] Edmunds LR, Sharma L, Kang A, Lu J, Vockley J, Basu S et al. c-Myc programs fatty acid metabolism and dictates acetyl-CoA abundance and fate. J Biol Chem 2014; 289: 25382–25392. 25053415 10.1074/jbc.M114.580662 PMC4155699

[bib104] Li X, Kazgan N. Mammalian sirtuins and energy metabolism. Int J Biol Sci 2011; 7: 575–587. 21614150 10.7150/ijbs.7.575 PMC3101526

[bib105] Fujiki R, Hashiba W, Sekine H, Yokoyama A, Chikanishi T, Ito S et al. GlcNAcylation of histone H2B facilitates its monoubiquitination. Nature 2011; 480: 557–560. 22121020 10.1038/nature10656 PMC7289526

[bib106] Sakabe K, Hart GW. O-GlcNAc transferase regulates mitotic chromatin dynamics. J Biol Chem 2010; 285: 34460–34468. 20805223 10.1074/jbc.M110.158170 PMC2966060

[bib107] Myers SA, Panning B, Burlingame AL. Polycomb repressive complex 2 is necessary for the normal site-specific O-GlcNAc distribution in mouse embryonic stem cells. Proc Natl Acad Sci USA 2011; 108: 9490–9495. 21606357 10.1073/pnas.1019289108 PMC3111310

[bib108] Fong JJ, Nguyen BL, Bridger R, Medrano EE, Wells L, Pan S et al. beta-N-Acetylglucosamine (O-GlcNAc) is a novel regulator of mitosis-specific phosphorylations on histone H3. J Biol Chem 2012; 287: 12195–12203. 22371497 10.1074/jbc.M111.315804 PMC3320971

[bib109] Chen Q, Chen Y, Bian C, Fujiki R, Yu X. TET2 promotes histone O-GlcNAcylation during gene transcription. Nature 2013; 493: 561–564. 23222540 10.1038/nature11742 PMC3684361

[bib110] Ito R, Katsura S, Shimada H, Tsuchiya H, Hada M, Okumura T et al. TET3-OGT interaction increases the stability and the presence of OGT in chromatin. Genes Cells 2014; 19: 52–65. 24304661 10.1111/gtc.12107

[bib111] Chu CS, Lo PW, Yeh YH, Hsu PH, Peng SH, Teng YC et al. O-GlcNAcylation regulates EZH2 protein stability and function. Proc Natl Acad Sci USA 2014; 111: 1355–1360. 24474760 10.1073/pnas.1323226111 PMC3910655

[bib112] Ranuncolo SM, Ghosh S, Hanover JA, Hart GW, Lewis BA. Evidence of the involvement of O-GlcNAc-modified human RNA polymerase II CTD in transcription*in vitro*and*in vivo*. J Biol Chem 2012; 287: 23549–23561. 22605332 10.1074/jbc.M111.330910 PMC3390630

[bib113] Pinho SS, Reis CA. Glycosylation in cancer: mechanisms and clinical implications. Nat Rev Cancer 2015; 15: 540–555. 26289314 10.1038/nrc3982

[bib114] Caldwell SA, Jackson SR, Shahriari KS, Lynch TP, Sethi G, Walker S et al. Nutrient sensor O-GlcNAc transferase regulates breast cancer tumorigenesis through targeting of the oncogenic transcription factor FoxM1. Oncogene 2010; 29: 2831–2842. 20190804 10.1038/onc.2010.41

[bib115] Lucena MC, Carvalho-Cruz P, Donadio JL, Oliveira IA, de Queiroz RM, Marinho-Carvalho MM et al. Epithelial Mesenchymal Transition Induces Aberrant Glycosylation through Hexosamine Biosynthetic Pathway Activation. J Biol Chem 2016; 291: 12917–12929. 27129262 10.1074/jbc.M116.729236 PMC4933211

[bib116] Onodera Y, Nam JM, Bissell MJ. Increased sugar uptake promotes oncogenesis via EPAC/RAP1 and O-GlcNAc pathways. J Clin Invest 2014; 124: 367–384. 24316969 10.1172/JCI63146 PMC3871217

[bib117] Itkonen HM, Minner S, Guldvik IJ, Sandmann MJ, Tsourlakis MC, Berge V et al. O-GlcNAc transferase integrates metabolic pathways to regulate the stability of c-MYC in human prostate cancer cells. Cancer Res 2013; 73: 5277–5287. 23720054 10.1158/0008-5472.CAN-13-0549

[bib118] Wellen KE, Lu C, Mancuso A, Lemons JM, Ryczko M, Dennis JW et al. The hexosamine biosynthetic pathway couples growth factor-induced glutamine uptake to glucose metabolism. Genes Dev 2010; 24: 2784–2799. 21106670 10.1101/gad.1985910 PMC3003197

[bib119] Guillaumond F, Leca J, Olivares O, Lavaut MN, Vidal N, Berthezène P et al. Strengthened glycolysis under hypoxia supports tumor symbiosis and hexosamine biosynthesis in pancreatic adenocarcinoma. Proc Natl Acad Sci USA 2013; 110: 3919–3924. 23407165 10.1073/pnas.1219555110 PMC3593894

[bib120] Liu X, Wang X, Zhang J, Lam EK, Shin VY, Cheng AS et al. Warburg effect revisited: an epigenetic link between glycolysis and gastric carcinogenesis. Oncogene 2010; 29: 442–450. 19881551 10.1038/onc.2009.332

[bib121] Chen M, Zhang J, Li N, Qian Z, Zhu M, Li Q et al. Promoter hypermethylation mediated downregulation of FBP1 in human hepatocellular carcinoma and colon cancer. PLoS One 2011; 6: e25564. 22039417 10.1371/journal.pone.0025564 PMC3198434

[bib122] Dong C, Yuan T, Wu Y, Wang Y, Fan TW, Miriyala S et al. Loss of FBP1 by Snail-mediated repression provides metabolic advantages in basal-like breast cancer. Cancer Cell 2013; 23: 316–331. 23453623 10.1016/j.ccr.2013.01.022 PMC3703516

[bib123] Li H, Wang J, Xu H, Xing R, Pan Y, Li W et al. Decreased fructose-1,6-bisphosphatase-2 expression promotes glycolysis and growth in gastric cancer cells. Mol Cancer 2013; 12: 110. 24063558 10.1186/1476-4598-12-110 PMC3849177

[bib124] Lopez-Serra P, Marcilla M, Villanueva A, Ramos-Fernandez A, Palau A, Leal L et al. A DERL3-associated defect in the degradation of SLC2A1 mediates the Warburg effect. Nat Commun 2014; 5: 3608. 24699711 10.1038/ncomms4608 PMC3988805

[bib125] Desai S, Ding M, Wang B, Lu Z, Zhao Q, Shaw K et al. Tissue-specific isoform switch and DNA hypomethylation of the pyruvate kinase PKM gene in human cancers. Oncotarget 2014; 5: 8202–8210. 24077665 10.18632/oncotarget.1159 PMC4226677

[bib126] Kang YH, Lee HS, Kim WH. Promoter methylation and silencing of PTEN in gastric carcinoma. Lab Invest 2002; 82: 285–291. 11896207 10.1038/labinvest.3780422

[bib127] Salvesen HB, MacDonald N, Ryan A, Jacobs IJ, Lynch ED, Akslen LA et al. PTEN methylation is associated with advanced stage and microsatellite instability in endometrial carcinoma. Int J Cancer 2001; 91: 22–26. 11149415 10.1002/1097-0215(20010101)91:1<22::aid-ijc1002>3.0.co;2-s

[bib128] Soria JC, Lee HY, Lee JI, Wang L, Issa JP, Kemp BL et al. Lack of PTEN expression in non-small cell lung cancer could be related to promoter methylation. Clin Cancer Res 2002; 8: 1178–1184. 12006535

[bib129] García JM, Silva J, Peña C, Garcia V, Rodríguez R, Cruz MA et al. Promoter methylation of the PTEN gene is a common molecular change in breast cancer. Genes Chromosomes Cancer 2004; 41: 117–124. 15287024 10.1002/gcc.20062

[bib130] Alvarez-Nuñez F, Bussaglia E, Mauricio D, Ybarra J, Vilar M, Lerma E et al. PTEN promoter methylation in sporadic thyroid carcinomas. Thyroid 2006; 16: 17–23. 16487009 10.1089/thy.2006.16.17

[bib131] Trojan J, Brieger A, Raedle J, Esteller M, Zeuzem S. 5'-CpG island methylation of the LKB1/STK11 promoter and allelic loss at chromosome 19p13.3 in sporadic colorectal cancer. Gut 2000; 47: 272–276. 10896921 10.1136/gut.47.2.272 PMC1727990

[bib132] Esteller M, Avizienyte E, Corn PG, Lothe RA, Baylin SB, Aaltonen LA et al. Epigenetic inactivation of LKB1 in primary tumors associated with the Peutz- Jeghers syndrome. Oncogene 2000; 19: 164–168. 10644993 10.1038/sj.onc.1203227

[bib133] Vanharanta S, Shu W, Brenet F, Hakimi AA, Heguy A, Viale A et al. Epigenetic expansion of VHL-HIF signal output drives multiorgan metastasis in renal cancer. Nat Med 2013; 19: 50–56. 23223005 10.1038/nm.3029 PMC3540187

[bib134] Herman JG, Latif F, Weng Y, Lerman MI, Zbar B, Liu S et al. Silencing of the VHL tumor-suppressor gene by DNA methylation in renal carcinoma. Proc Natl Acad Sci USA 1994; 91: 9700–9704. 7937876 10.1073/pnas.91.21.9700 PMC44884

[bib135] Schmitt AM, Schmid S, Rudolph T, Anlauf M, Prinz C, Klöppel G et al. VHL inactivation is an important pathway for the development of malignant sporadic pancreatic endocrine tumors. Endocr Relat Cancer 2009; 16: 1219–1227. 19690016 10.1677/ERC-08-0297

[bib136] Hatzimichael E, Dasoula A, Shah R, Syed N, Papoudou-Bai A, Coley HM et al. The prolyl-hydroxylase EGLN3 and not EGLN1 is inactivated by methylation in plasma cell neoplasia. Eur J Haematol 2010; 84: 47–51. 19737309 10.1111/j.1600-0609.2009.01344.x

[bib137] Rawluszko AA, Bujnicka KE, Horbacka K, Krokowicz P, Jagodzinski PP. Expression and DNA methylation levels of prolyl hydroxylases PHD1, PHD2, PHD3 and asparaginyl hydroxylase FIH in colorectal cancer. BMC Cancer 2013; 13: 526. 24195777 10.1186/1471-2407-13-526 PMC3828400

[bib138] Place TL, Fitzgerald MP, Venkataraman S, Vorrink SU, Case AJ, Teoh ML et al. Aberrant promoter CpG methylation is a mechanism for impaired PHD3 expression in a diverse set of malignant cells. PLoS ONE 2011; 6: e14617. 21297970 10.1371/journal.pone.0014617 PMC3030558

[bib139] Xiao C, Kim HS, Lahusen T, Wang RH, Xu X, Gavrilova O et al. SIRT6 deficiency results in severe hypoglycemia by enhancing both basal and insulin-stimulated glucose uptake in mice. J Biol Chem 2010; 285: 36776–36784. 20847051 10.1074/jbc.M110.168039 PMC2978606

[bib140] Zhong L, D'Urso A, Toiber D, Sebastian C, Henry RE, Vadysirisack DD et al. The histone deacetylase Sirt6 regulates glucose homeostasis via Hif1alpha. Cell 2010; 140: 280–293. 20141841 10.1016/j.cell.2009.12.041 PMC2821045

[bib141] Zhong L, Mostoslavsky R. SIRT6: a master epigenetic gatekeeper of glucose metabolism. Transcription 2010; 1: 17–21. 21327158 10.4161/trns.1.1.12143 PMC3035182

[bib142] Sebastián C, Zwaans BM, Silberman DM, Gymrek M, Goren A, Zhong L et al. The histone deacetylase SIRT6 is a tumor suppressor that controls cancer metabolism. Cell 2012; 151: 1185–1199. 23217706 10.1016/j.cell.2012.10.047 PMC3526953

[bib143] Zhang ZG, Qin CY. Sirt6 suppresses hepatocellular carcinoma cell growth via inhibiting the extracellular signalregulated kinase signaling pathway. Mol Med Rep 2014; 9: 882–888. 24366394 10.3892/mmr.2013.1879

[bib144] Shin J, He M, Liu Y, Paredes S, Villanova L, Brown K et al. SIRT7 represses Myc activity to suppress ER stress and prevent fatty liver disease. Cell Rep 2013; 5: 654–665. 24210820 10.1016/j.celrep.2013.10.007 PMC3888240

[bib145] Barber MF, Michishita-Kioi E, Xi Y, Tasselli L, Kioi M, Moqtaderi Z et al. SIRT7 links H3K18 deacetylation to maintenance of oncogenic transformation. Nature 2012; 487: 114–118. 22722849 10.1038/nature11043 PMC3412143

[bib146] Liu PY, Xu N, Malyukova A, Scarlett CJ, Sun YT, Zhang XD et al. The histone deacetylase SIRT2 stabilizes Myc oncoproteins. Cell Death Differ 2013; 20: 503–514. 23175188 10.1038/cdd.2012.147 PMC3569991

[bib147] Yamasaki T, Seki N, Yoshino H, Itesako T, Yamada Y, Tatarano S et al. Tumor- suppressive microRNA-1291 directly regulates glucose transporter 1 in renal cell carcinoma. Cancer Sci 2013; 104: 1411–1419. 23889809 10.1111/cas.12240 PMC7654250

[bib148] Fei X, Qi M, Wu B, Song Y, Wang Y, Li T et al. MicroRNA-195-5p suppresses glucose uptake and proliferation of human bladder cancer T24 cells by regulating GLUT3 expression. FEBS Lett 2012; 586: 392–397. 22265971 10.1016/j.febslet.2012.01.006

[bib149] Dai DW, Lu Q, Wang LX, Zhao WY, Cao YQ, Li YN et al. Decreased miR-106a inhibits glioma cell glucose uptake and proliferation by targeting SLC2A3 in GBM. BMC cancer 2013; 13: 478. 24124917 10.1186/1471-2407-13-478 PMC3853007

[bib150] Chen YH, Heneidi S, Lee JM, Layman LC, Stepp DW, Gamboa GM et al. miRNA-93 inhibits GLUT4 and is overexpressed in adipose tissue of polycystic ovary syndrome patients and women with insulin resistance. Diabetes 2013; 62: 2278–2286. 23493574 10.2337/db12-0963 PMC3712080

[bib151] Peschiaroli A, Giacobbe A, Formosa A, Markert EK, Bongiorno-Borbone L, Levine AJ et al. miR-143 regulates hexokinase 2 expression in cancer cells. Oncogene 2013; 32: 797–802. 22469988 10.1038/onc.2012.100

[bib152] Fang R, Xiao T, Fang Z, Sun Y, Li F, Gao Y et al. MicroRNA-143 (miR-143) regulates cancer glycolysis via targeting hexokinase 2 gene. J Biol Chem 2012; 287: 23227–23235. 22593586 10.1074/jbc.M112.373084 PMC3391126

[bib153] Jiang S, Zhang LF, Zhang HW, Hu S, Lu MH, Liang S et al. A novel miR-155/miR-143 cascade controls glycolysis by regulating hexokinase 2 in breast cancer cells. EMBO J 2012; 31: 1985–1998. 22354042 10.1038/emboj.2012.45 PMC3343331

[bib154] Gregersen LH, Jacobsen A, Frankel LB, Wen J, Krogh A, Lund AH et al. MicroRNA-143 down-regulates Hexokinase 2 in colon cancer cells. BMC cancer 2012; 12: 232. 22691140 10.1186/1471-2407-12-232 PMC3480834

[bib155] Zhao S, Liu H, Liu Y, Wu J, Wang C, Hou X et al. miR-143 inhibits glycolysis and depletes stemness of glioblastoma stem-like cells. Cancer Lett 2013; 333: 253–260. 23376635 10.1016/j.canlet.2013.01.039

[bib156] Yoshino H, Enokida H, Itesako T, Kojima S, Kinoshita T, Tatarano S et al. Tumor-suppressive microRNA-143/145 cluster targets hexokinase-2 in renal cell carcinoma. Cancer Sci 2013; 104: 1567–1574. 24033605 10.1111/cas.12280 PMC7653528

[bib157] Ahmad A, Aboukameel A, Kong D, Wang Z, Sethi S, Chen W et al. Phosphoglucose isomerase/autocrine motility factor mediates epithelial- mesenchymal transition regulated by miR-200 in breast cancer cells. Cancer Res 2011; 71: 3400–3409. 21389093 10.1158/0008-5472.CAN-10-0965 PMC3085607

[bib158] Castoldi M, Vujic Spasic M, Altamura S, Elmén J, Lindow M, Kiss J et al. The liver-specific microRNA miR-122 controls systemic iron homeostasis in mice. J Clin Invest 2011; 121: 1386–1396. 21364282 10.1172/JCI44883 PMC3069782

[bib159] Kefas B, Comeau L, Erdle N, Montgomery E, Amos S, Purow B. Pyruvate kinase M2 is a target of the tumor-suppressive microRNA-326 and regulates the survival of glioma cells. Neuro Oncol 2010; 12: 1102–1112. 20667897 10.1093/neuonc/noq080 PMC3098027

[bib160] Wong TS, Liu XB, Ho CW, Yuen PW, Ng WM, Wei IW. Identification of pyruvate kinase type M2 as potential oncoprotein in squamous cell carcinoma of tongue through microRNA profiling. Int J Cancer 2008; 123: 251–257. 18464261 10.1002/ijc.23583

[bib161] Rathore MG, Saumet A, Rossi JF, de Bettignies C, Tempé D, Lecellier CH et al. The NF-kappaB member p65 controls glutamine metabolism through miR-23a. Int J Biochem Cell Biol 2012; 44: 1448–1456. 22634383 10.1016/j.biocel.2012.05.011

[bib162] Chan SY, Zhang YY, Hemann C, Mahoney CE, Zweier JL, Loscalzo J. MicroRNA-210 controls mitochondrial metabolism during hypoxia by repressing the iron-sulfur cluster assembly proteins ISCU1/2. Cell Metab 2009; 10: 273–284. 19808020 10.1016/j.cmet.2009.08.015 PMC2759401

[bib163] Cluntun AA, Huang H, Dai L, Liu X, Zhao Y, Locasale JW. The rate of glycolysis quantitatively mediates specific histone acetylation sites. Cancer Metab 2015; 3: 10. 26401273 10.1186/s40170-015-0135-3 PMC4579576

[bib164] Liu XS, Little JB, Yuan ZM. Glycolytic metabolism influences global chromatin structure. Oncotarget 2015; 6: 4214–4225. 25784656 10.18632/oncotarget.2929 PMC4414184

[bib165] Chen W, Gueron M. The inhibition of bovine heart hexokinase by 2-deoxy-D-glucose-6-phosphate: characterization by 31 P NMR and metabolic implications. Biochimie 1992; 74: 867–873. 1467345 10.1016/0300-9084(92)90070-u

[bib166] Moussaieff A, Rouleau M, Kitsberg D, Cohen M, Levy G, Barasch D et al. Glycolysis-mediated changes in acetyl-CoA and histone acetylation control the early differentiation of embryonic stem cells. Cell Metab 2015; 21: 392–402. 25738455 10.1016/j.cmet.2015.02.002

[bib167] Robinson MM, McBryant SJ, Tsukamoto T, Rojas C, Ferraris DV, Hamilton SK et al. Novel mechanism of inhibition of rat kidney-type glutaminase by bis-2-(5-phenylacetamido-1,2,4-thiadiazol-2-yl)ethyl sulfide (BPTES). Biochem J 2007; 406: 407–414. 17581113 10.1042/BJ20070039 PMC2049044

[bib168] Wang JB, Erickson JW, Fuji R, Ramachandran S, Gao P, Dinavahi R et al. Targeting mitochondrial glutaminase activity inhibits oncogenic transformation. Cancer Cell 2010; 18: 207–219. 20832749 10.1016/j.ccr.2010.08.009 PMC3078749

[bib169] Simpson NE, Tryndyak VP, Pogribna M, Beland FA, Pogribny IP. Modifying metabolically sensitive histone marks by inhibiting glutamine metabolism affects gene expression and alter cancer cell phenotype. Epigenetics 2012; 7: 1413–1420. 23117580 10.4161/epi.22713 PMC3528696

[bib170] Simpson NE, Tryndyak VP, Beland FA, Pogribny IP. An*in vitro*investigation of metabolically sensitive biomarkers in breast cancer progression. Breast Cancer Res Treat 2012; 133: 959–968. 22101407 10.1007/s10549-011-1871-x

[bib171] Elhammali A, Ippolito JE, Collins L, Crowley J, Marasa J, Piwnica-Worms D. A high-throughput fluorimetric assay for 2-hydroxyglutarate identifies Zaprinast as a glutaminase inhibitor. Cancer Discov 2014; 4: 828–839. 24740997 10.1158/2159-8290.CD-13-0572 PMC4197823

[bib172] Rohle D1, Popovici-Muller J, Palaskas N, Turcan S, Grommes C, Campos C et al. An inhibitor of mutant IDH1 delays growth and promotes differentiation of glioma cells. Science 2013; 340: 626–630. 23558169 10.1126/science.1236062 PMC3985613

[bib173] Li L, Paz AC, Wilky BA, Johnson B, Galoian K, Rosenberg A et al. Treatment with a small molecule mutant IDH1 inhibitor suppresses tumorigenic activity and decreases production of the oncometabolite 2-hydroxyglutarate in human chondrosarcoma cells. PLoS One 2015; 10: e0133813. 26368816 10.1371/journal.pone.0133813 PMC4569544

[bib174] Davis MI, Gross S, Shen M, Straley KS, Pragani R, Lea WA et al. Biochemical, cellular, and biophysical characterization of a potent inhibitor of mutant isocitrate dehydrogenase IDH1. J Biol Chem 2014; 289: 13717–13725. 24668804 10.1074/jbc.M113.511030 PMC4022846

[bib175] Kim HJ, Choi BY, Keum YS. Identification of a new selective chemical inhibitor of mutant isocitrate dehydrogenase-1. J Cancer Prev 2015; 20: 78–83. 25853107 10.15430/JCP.2015.20.1.78 PMC4384718

[bib176] Deng G, Shen J, Yin M, McManus J, Mathieu M, Gee P et al. Selective inhibition of mutant isocitrate dehydrogenase 1 (IDH1) via disruption of a metal binding network by an allosteric small molecule. J Biol Chem 2015; 290: 762–774. 25391653 10.1074/jbc.M114.608497 PMC4294499

[bib177] Zheng B, Yao Y, Liu Z, Deng L, Anglin JL, Jiang H et al. Crystallographic investigation and selective inhibition of mutant isocitrate dehydrogenase. ACS Med Chem Lett 2013; 4: 542–546. 23795241 10.1021/ml400036z PMC3686309

[bib178] Okoye-Okafor UC, Bartholdy B, Cartier J, Gao EN, Pietrak B, Rendina AR et al. New IDH1 mutant inhibitors for treatment of acute myeloid leukemia. Nat Chem Biol 2015; 11: 878–886. 26436839 10.1038/nchembio.1930 PMC5155016

[bib179] Shih AH, Shank KR, Meydan C, Intlekofer AM, Ward P, Thompson CB et al. AG-221, a small molecule mutant IDH2 inhibitor, remodels epigenetic state of IDH2-mutant cells and induces alterations in self-renewal/differentiation in IDH2-mutant AML model*in vivo*. Blood 2014; 124: 237.

[bib180] Ellwood-Yen K, Wang F, Travins J, Chen Y, Yang H, Straley K et al. AG-221 offers a survival advantage in a primary human IDH2 mutant AML xenograft model. Cancer Res 2014; 74(Suppl): 3116.

[bib181] Yen K, Wang F, Travins J, Chen Y, Yang H, Straley K et al. AG-221 offers a survival advantage in a primary human IDH2 mutant AML xenograft model. Blood 2013; 122: 240.

[bib182] DiNardo C, Stein EM, Altman JK, Collins R, DeAngelo DJ, Fathi AT et al. Ag-221, an oral, selective, first-in-class, potent inhibitor of the IDH2 mutant enzyme, induced durable responses in a phase 1 study of IDH2 mutation-positive advanced hematologic malignancies. Haematologica 2015; 100: 216–217.

[bib183] Wang F, Travins J, DeLaBarre B, Penard-Lacronique V, Schalm S, Hansen E et al. Targeted inhibition of mutant IDH2 in leukemia cells induces cellular differentiation. Science 2013; 340: 622–626. 23558173 10.1126/science.1234769

[bib184] Kernytsky A, Wang F, Hansen E, Schalm S, Straley K, Gliser C et al. IDH2 mutation-induced histone and DNA hypermethylation is progressively reversed by small-molecule inhibition. Blood 2015; 125: 296–303. 25398940 10.1182/blood-2013-10-533604 PMC4295919

[bib185] Glazer RI, Hartman KD, Knode MC, Richard MM, Chiang PK, Tseng CK et al. 3-Deazaneplanocin: a new and potent inhibitor of S-adenosylhomocysteine hydrolase and its effects on human promyelocytic leukemia cell line HL-60. Biochem Biophys Res Commun 1986; 135: 688–694. 3457563 10.1016/0006-291x(86)90048-3

[bib186] Miranda TB, Cortez CC, Yoo CB, Liang G, Abe M, Kelly TK et al. DZNep is a global histone methylation inhibitor that reactivates developmental genes not silenced by DNA methylation. Mol Cancer Ther 2009; 8: 1579–1588. 19509260 10.1158/1535-7163.MCT-09-0013 PMC3186068

[bib187] Momparler RL, Cote S, Momparler LF, Idaghdour Y. Epigenetic therapy of acute myeloid leukemia using 5-aza-2'-deoxycytidine (decitabine) in combination with inhibitors of histone methylation and deacetylation. Clin Epigenet 2014; 6: 19. 10.1186/1868-7083-6-19 PMC4194463 25313314

[bib188] Momparler RL, Idaghdour Y, Marquez VE, Momparler LF. Synergistic antileukemic action of a combination of inhibitors of DNA methylation and histone methylation. Leuk Res 2012; 36: 1049–1054. 22472464 10.1016/j.leukres.2012.03.001

[bib189] Jiang X, Tan J, Li J, Kivimäe S, Yang X, Zhuang L et al. DACT3 is an epigenetic regulator of Wnt/beta-catenin signaling in colorectal cancer and is a therapeutic target of histone modifications. Cancer Cell 2008; 13: 529–541. 18538736 10.1016/j.ccr.2008.04.019 PMC2577847

[bib190] Momparler RL, Cote S. Targeting of cancer stem cells by inhibitors of DNA and histone methylation. Expert Opin Investig Drugs 2015; 24: 1031–1043. 10.1517/13543784.2015.1051220 26004134

[bib191] Kraus D, Yang Q, Kong D, Banks AS, Zhang L, Rodgers JT et al. Nicotinamide N-methyltransferase knockdown protects against diet-induced obesity. Nature 2014; 508: 258–262. 24717514 10.1038/nature13198 PMC4107212

[bib192] Dehennaut V, Lefebvre T, Sellier C, Leroy Y, Gross B, Walker S et al. O-linked N-acetylglucosaminyltransferase inhibition prevents G2/M transition in Xenopus laevis oocytes. J Biol Chem 2007; 282: 12527–12536. 17329255 10.1074/jbc.M700444200

[bib193] Olivier-Van Stichelen S, Guinez C, Mir AM, Perez-Cervera Y, Liu C, Michalski JC et al. The hexosamine biosynthetic pathway and O-GlcNAcylation drive the expression of beta-catenin and cell proliferation. Am J Physiol Endocrinol Metab 2012; 302: E417–E424. 22114026 10.1152/ajpendo.00390.2011

[bib194] Zhou F, Huo J, Liu Y, Liu H, Liu G, Chen Y et al. Elevated glucose levels impair the WNT/beta-catenin pathway via the activation of the hexosamine biosynthesis pathway in endometrial cancer. J Steroid Biochem Mol Biol 2016; 159: 19–25. 26923859 10.1016/j.jsbmb.2016.02.015

[bib195] Olivier-Van Stichelen S, Dehennaut V, Buzy A, Zachayus JL, Guinez C, Mir AM et al. O-GlcNAcylation stabilizes beta-catenin through direct competition with phosphorylation at threonine 41. FASEB J 2014; 28: 3325–3338. 24744147 10.1096/fj.13-243535 PMC4101651

[bib196] Lin SH, Liu T, Ming X, Tang Z, Fu L, Schmitt-Kopplin P et al. Regulatory role of hexosamine biosynthetic pathway on hepatic cancer stem cell marker CD133 under low glucose conditions. Sci Rep 2016; 6: 21184. 26878908 10.1038/srep21184 PMC4754761

[bib197] Rodriguez-Paredes M, Esteller M. A combined epigenetic therapy equals the efficacy of conventional chemotherapy in refractory advanced non-small cell lung cancer. Cancer Discov 2011; 1: 557–559. 22586680 10.1158/2159-8290.CD-11-0271

[bib198] Borodovsky A, Salmasi V, Turcan S, Fabius AW, Baia GS, Eberhart CG et al. 5-azacytidine reduces methylation, promotes differentiation and induces tumor regression in a patient-derived IDH1 mutant glioma xenograft. Oncotarget 2013; 4: 1737–1747. 24077805 10.18632/oncotarget.1408 PMC3858560

[bib199] Turcan S, Fabius AW, Borodovsky A, Pedraza A, Brennan C, Huse J et al. Efficient induction of differentiation and growth inhibition in IDH1 mutant glioma cells by the DNMT inhibitor decitabine. Oncotarget 2013; 4: 1729–1736. 24077826 10.18632/oncotarget.1412 PMC3858559

[bib200] Alcarraz-Vizan G, Boren J, Lee WN, Cascante M. Histone deacetylase inhibition results in a common metabolic profile associated with HT29 differentiation. Metabolomics 2010; 6: 229–237. 20445757 10.1007/s11306-009-0192-0 PMC2862949

[bib201] Wardell SE, Ilkayeva OR, Wieman HL, Frigo DE, Rathmell JC, Newgard CB et al. Glucose metabolism as a target of histone deacetylase inhibitors. Mol Endocrinol 2009; 23: 388–401. 19106193 10.1210/me.2008-0179 PMC2654518

[bib202] Amoêdo ND, Rodrigues MF, Pezzuto P, Galina A, da Costa RM, de Almeida FC et al. Energy metabolism in H460 lung cancer cells: effects of histone deacetylase inhibitors. PLoS One 2011; 6: e22264. 21789245 10.1371/journal.pone.0022264 PMC3138778

[bib203] Rodrigues MF, Carvalho E, Pezzuto P, Rumjanek FD, Amoedo ND. Reciprocal modulation of histone deacetylase inhibitors sodium butyrate and trichostatin A on the energy metabolism of breast cancer cells. J Cell Biochem 2015; 116: 797–808. 25510910 10.1002/jcb.25036

[bib204] Feldman JL, Baeza J, Denu JM. Activation of the protein deacetylase SIRT6 by long-chain fatty acids and widespread deacylation by mammalian sirtuins. J Biol Chem 2013; 288: 31350–31356. 24052263 10.1074/jbc.C113.511261 PMC3829447

[bib205] Villalba JM, Alcain FJ. Sirtuin activators and inhibitors. BioFactors 2012; 38: 349–359. 22730114 10.1002/biof.1032 PMC3467333

[bib206] Bader AG, Brown D, Winkler M. The promise of microRNA replacement therapy. Cancer Res 2010; 70: 7027–7030. 20807816 10.1158/0008-5472.CAN-10-2010 PMC2940943

[bib207] Ebert MS, Sharp PA. MicroRNA sponges: progress and possibilities. RNA 2010; 16: 2043–2050. 20855538 10.1261/rna.2414110 PMC2957044

[bib208] Li Z, Rana TM. Therapeutic targeting of microRNAs: current status and future challenges. Nat Rev Drug Discov 2014; 13: 622–638. 25011539 10.1038/nrd4359

[bib209] Meng F, Henson R, Wehbe-Janek H, Ghoshal K, Jacob ST, Patel T. MicroRNA-21 regulates expression of the PTEN tumor suppressor gene in human hepatocellular cancer. Gastroenterology 2007; 133: 647–658. 17681183 10.1053/j.gastro.2007.05.022 PMC4285346

[bib210] Pereira DM, Rodrigues PM, Borralho PM, Rodrigues CM. Delivering the promise of miRNA cancer therapeutics. Drug Discov Today 2013; 18: 282–289. 23064097 10.1016/j.drudis.2012.10.002

[bib211] Braiteh F, Soriano AO, Garcia-Manero G, Hong D, Johnson MM, Silva Lde P et al. Phase I study of epigenetic modulation with 5-azacytidine and valproic acid in patients with advanced cancers. Clin Cancer Res 2008; 14: 6296–6301. 18829512 10.1158/1078-0432.CCR-08-1247 PMC2582814

[bib212] Juergens RA, Wrangle J, Vendetti FP, Murphy SC, Zhao M, Coleman B et al. Combination epigenetic therapy has efficacy in patients with refractory advanced non-small cell lung cancer. Cancer Discov 2011; 1: 598–607. 22586682 10.1158/2159-8290.CD-11-0214 PMC3353724

[bib213] Chu BF, Karpenko MJ, Liu Z, Aimiuwu J, Villalona-Calero MA, Chan KK et al. Phase I study of 5-aza-2'-deoxycytidine in combination with valproic acid in non-small-cell lung cancer. Cancer Chemother Pharmacol 2013; 71: 115–121. 23053268 10.1007/s00280-012-1986-8

[bib214] Brown JM, Giaccia AJ. The unique physiology of solid tumors: opportunities (and problems) for cancer therapy. Cancer Res 1998; 58: 1408–1416. 9537241

[bib215] Wilson WR, Hay MP. Targeting hypoxia in cancer therapy. Nat Rev Cancer 2011; 11: 393–410. 21606941 10.1038/nrc3064

[bib216] Johnson AB, Denko N, Barton MC. Hypoxia induces a novel signature of chromatin modifications and global repression of transcription. Mutat Res 2008; 640: 174–179. 18294659 10.1016/j.mrfmmm.2008.01.001 PMC2346607

[bib217] Skowronski K, Dubey S, Rodenhiser D, Coomber B. Ischemia dysregulates DNA methyltransferases and p16INK4a methylation in human colorectal cancer cells. Epigenetics 2010; 5: 547–556. 20543577 10.4161/epi.5.6.12400 PMC3322492

[bib218] Liu Q, Liu L, Zhao Y, Zhang J, Wang D, Chen J et al. Hypoxia induces genomic DNA demethylation through the activation of HIF-1α and transcriptional upregulation of MAT2A in hepatoma cells. Mol Cancer Ther 2011; 10: 1113–1123. 21460102 10.1158/1535-7163.MCT-10-1010

[bib219] Mariani CJ, Vasanthakumar A, Madzo J, Yesilkanal A, Bhagat T, Yu Y et al. TET1-mediated hydroxymethylation facilitates hypoxic gene induction in neuroblastoma. Cell Rep 2014; 7: 1343–1352. 24835990 10.1016/j.celrep.2014.04.040 PMC4516227

[bib220] Hattori M, Yokoyama Y, Hattori T, Motegi S, Amano H, Hatada I et al. Global DNA hypomethylation and hypoxia-induced expression of the ten eleven translocation (TET) family, TET1, in scleroderma fibroblasts. Exp Dermatol 2015; 24: 841–846. 26013976 10.1111/exd.12767

[bib221] Lee SH, Kim J, Kim WH, Lee YM. Hypoxic silencing of tumor suppressor RUNX3 by histone modification in gastric cancer cells. Oncogene 2009; 28: 184–194. 18850007 10.1038/onc.2008.377

[bib222] Daud AI, Dawson J, DeConti RC, Bicaku E, Marchion D, Bastien S et al. Potentiation of a topoisomerase I inhibitor, karenitecin, by the histone deacetylase inhibitor valproic acid in melanoma: translational and phase I/II clinical trial. Clin Cancer Res 2009; 15: 2479–2487. 19318485 10.1158/1078-0432.CCR-08-1931

[bib223] Ramalingam SS, Maitland ML, Frankel P, Argiris AE, Koczywas M, Gitlitz B et al. Carboplatin and Paclitaxel in combination with either vorinostat or placebo for first-line therapy of advanced non-small-cell lung cancer. J Clin Oncol 2010; 28: 56–62. 19933908 10.1200/JCO.2009.24.9094 PMC2799233

[bib224] Munster PN, Thurn KT, Thomas S, Raha P, Lacevic M, Miller A et al. A phase II study of the histone deacetylase inhibitor vorinostat combined with tamoxifen for the treatment of patients with hormone therapy-resistant breast cancer. Br J Cancer 2011; 104: 1828–1835. 21559012 10.1038/bjc.2011.156 PMC3111195

[bib225] Witta SE, Jotte RM, Konduri K, Neubauer MA, Spira AI, Ruxer RL et al. Randomized phase II trial of erlotinib with and without entinostat in patients with advanced non-small-cell lung cancer who progressed on prior chemotherapy. J Clin Oncol 2012; 30: 2248–2255. 22508830 10.1200/JCO.2011.38.9411 PMC4798782

